# M4-like Macrophages Drive Skeletal Muscle Fibrosis and Suppress Myogenesis via the MMP12/SPP1/NF-κB Axis

**DOI:** 10.3390/ijms27209369

**Published:** 2026-10-21

**Authors:** Senwen Yao, Zhenyuan Lu, Guosheng Zhao, Yang Liu, Shan Wu, Jiaxuan Zhang, Renjie Dong, Yangming Hu, Ye Zhang, Yang Wang

**Affiliations:** Department of Orthopedics, The Second Affiliated Hospital of Chongqing Medical University, Yuzhong, Chongqing 400010, China

**Keywords:** skeletal muscle injury, M4-like macrophage, fibrosis, SPP1, MMP12, skeletal muscle repair

## Abstract

Acute skeletal muscle repair depends on a finely tuned balance between myogenic regeneration and fibrotic remodeling. Macrophages are essential to this process, and specific subsets have been linked to fibrosis in some organs. The M4-like macrophage, a distinct polarization subset implicated in fibrotic diseases, has not been well characterized in the context of injured muscle, particularly regarding its impact on fibroblast activation and myogenic suppression. The study aims to explore the effects of M4-like macrophages in the repair of muscle injury and possible mechanism. We analyzed a murine single-cell RNA-sequencing dataset from cardiotoxin (CTX)-injured skeletal muscle to identify injury-expanded macrophage subsets and their molecular programs. Immunohistochemistry and immunofluorescence staining of muscle sections were used to assess CXCL4 expression and platelet (CD41^+^) infiltration in injured versus uninjured tissues. CXCL4-induced M4-like macrophages were established as an in vitro model to examine their effects on L929 fibroblasts and C2C12 myoblasts. The MMP12–SPP1 interaction was predicted by AlphaFold3 and validated by co-immunoprecipitation and immunofluorescence colocalization. Loss-of-function and rescue experiments targeting MMP12 and SPP1 were performed to determine their causal roles in fibroblast activation and myogenic suppression. Transcriptomic and biochemical analyses were used to define downstream macrophage-intrinsic NF-κB/TNF-α signaling. Finally, AAV-mediated macrophage-targeted *SPP1* knockdown and pharmacological NF-κB inhibition were evaluated in the CTX injury model. Using a BALB/c mouse model of cardiotoxin (CTX)-induced acute skeletal muscle injury and a publicly available murine scRNA-seq dataset from CTX-injured skeletal muscle, we observed an injury-induced *MMP12/SPP1*-positive macrophage subset with an M4-like transcriptional signature. Mechanistically, co-immunoprecipitation assays and immunofluorescence colocalization supported an association between MMP12 and SPP1. *MMP12* knockdown attenuated the pro-fibrotic and anti-myogenic effects of M4-like macrophages, whereas recombinant SPP1 largely restored these phenotypes. Furthermore, transcriptomic and biochemical analyses revealed that autocrine SPP1 activated NF-κB signaling in M4-like macrophages, and increased TNF-α secretion. Pharmacological inhibition of NF-κB signaling with QNZ reduced fibroblast activation and restored the expression of myogenic markers. However, QNZ is not TNF-α specific and may affect multiple cell types. In vivo, AAV-mediated macrophage-targeted *SPP1* knockdown driven by the F4/80 promoter, as well as QNZ treatment in the CTX-induced muscle injury model, markedly decreased collagen deposition, enhanced myogenic regeneration, and improved functional recovery. These findings identify an MMP12/SPP1/NF-κB signaling pathway in M4-like macrophages, with TNF-α as a potential downstream mediator, that may contribute to an imbalance between regeneration and fibrosis and may represent a therapeutic target for enhancing skeletal muscle repair after acute injury.

## 1. Introduction

Skeletal muscle has a remarkable capacity for regeneration following acute injury. This repair process depends on a tightly coordinated sequence of inflammation, activation and differentiation of myogenic precursor cells, and tissue remodeling [1,2,3]. The immune system, particularly macrophages, plays a pivotal role in orchestrating these phases. Initially, pro-inflammatory (M1-like) macrophages clear necrotic debris and release signals that activate satellite cells. Subsequently, macrophages transition toward a more regulatory and pro-regenerative state, often broadly described as an M2-like phenotype [4,5,6]. However, failure of this timely phenotypic transition, or aberrant activation of pro-fibrotic macrophage programs, can disrupt the balance between regeneration and fibrosis, leading to excessive extracellular matrix (ECM) deposition, impaired myofiber regeneration, and long-term functional impairment [7,8,9,10,11].

Single-cell RNA sequencing has revealed extensive macrophage heterogeneity in injured tissues [12,13,14,15]. Among these, *SPP1*^+^ macrophages are established drivers of fibrosis across multiple organs. Yet the upstream molecule events enabling SPP1 function, particularly in skeletal muscle, remain unclear. Among *SPP1*-expressing subsets, a particular population referred to as M4-like macrophages characterized by high co-expression of *MMP12* has been implicated in fibrotic disorders. However, whether this coordinated *MMP12-SPP1* expression reflects a functional cooperation that drives skeletal muscle repair remains unknown.

In this study, we integrated single-cell transcriptomics with in vitro and in vivo functional models to investigate how macrophages regulate the balance between regeneration and fibrosis during acute skeletal muscle repair. In the cardiotoxin (CTX)-induced acute skeletal muscle injury model, we observed an injury-expanded *MMP12/SPP1*-positive macrophage subset with an M4-like transcriptional signature. We further investigated the interaction between MMP12 and SPP1 as a key mechanism by which these macrophages promote fibroblast activation and inhibit myogenic differentiation. Mechanistically, this interaction activated autocrine NF-κB signaling in M4-like macrophages and enhanced TNF-α secretion, which may contribute to fibrogenesis and suppression of myogenesis. Collectively, our findings identify an MMP12/SPP1/NF-κB signaling axis in M4-like macrophages, with TNF-α as a potential downstream mediator, that may regulate the regeneration–fibrosis balance and may represent a potential therapeutic target for improving skeletal muscle repair after acute injury.

## 2. Results

### 2.1. CTX-Induced Microenvironmental Remodeling and the Pseudotime Trajectory of MMP12/SPP1-Positive Macrophages Subset with an M4-like Transcriptional Signature

**Figure 1:**
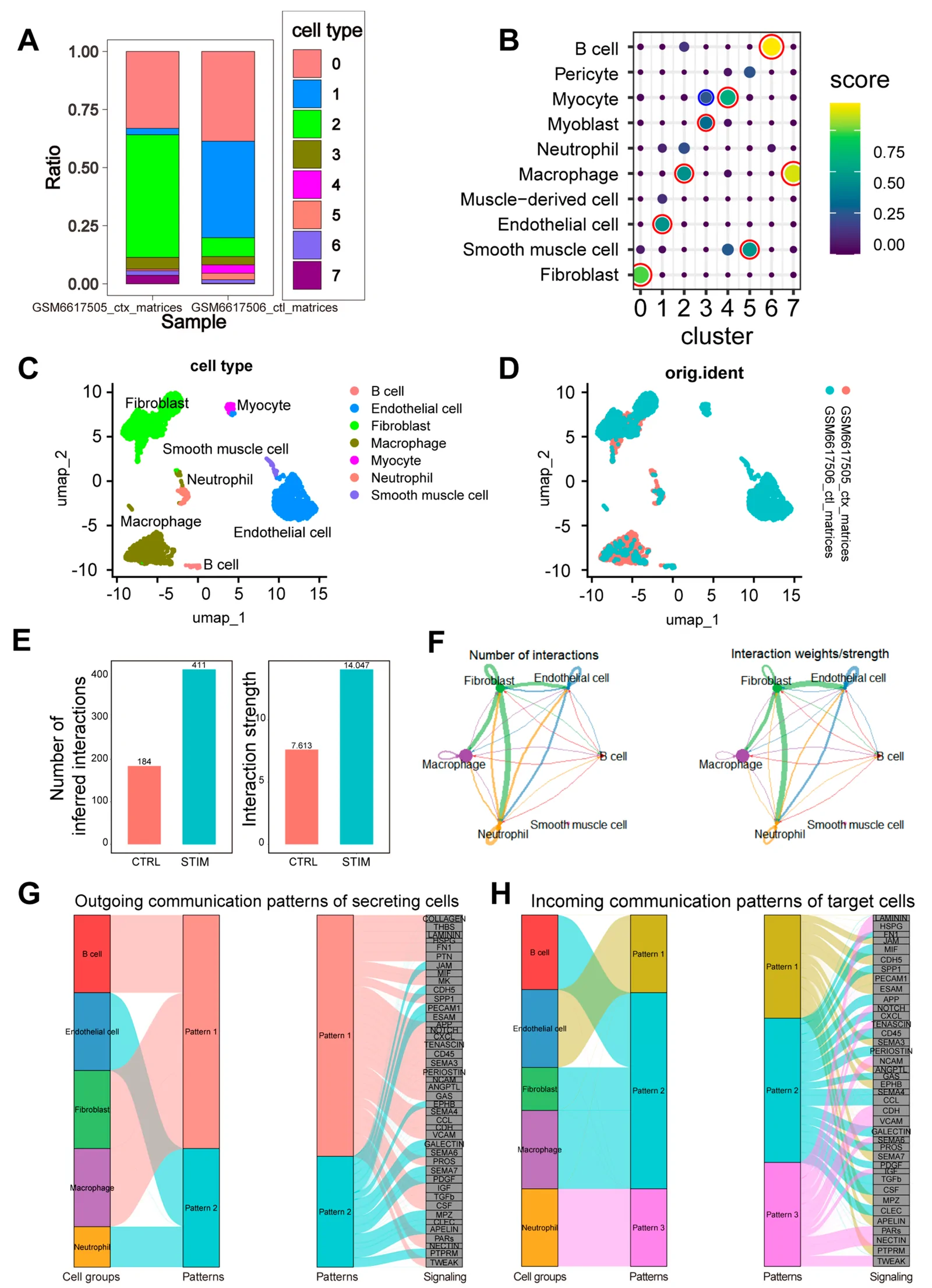
Cell type annotation and clustering of single-cell transcriptomes from CTX-injured and control skeletal muscle. (**A**) The ratio of different cell types in the CTX-induced injury and control muscle sample. (**B**) Cell annotation in the muscle samples. (**C**,**D**) Cellular umap grouping of the CTX-induced injury and control muscle sample. (**E**,**F**) Analysis of the interactions in the muscle samples. (**G**,**H**) Identification and visualization of efferent signaling communication patterns from secretory cells.

**Figure 2:**
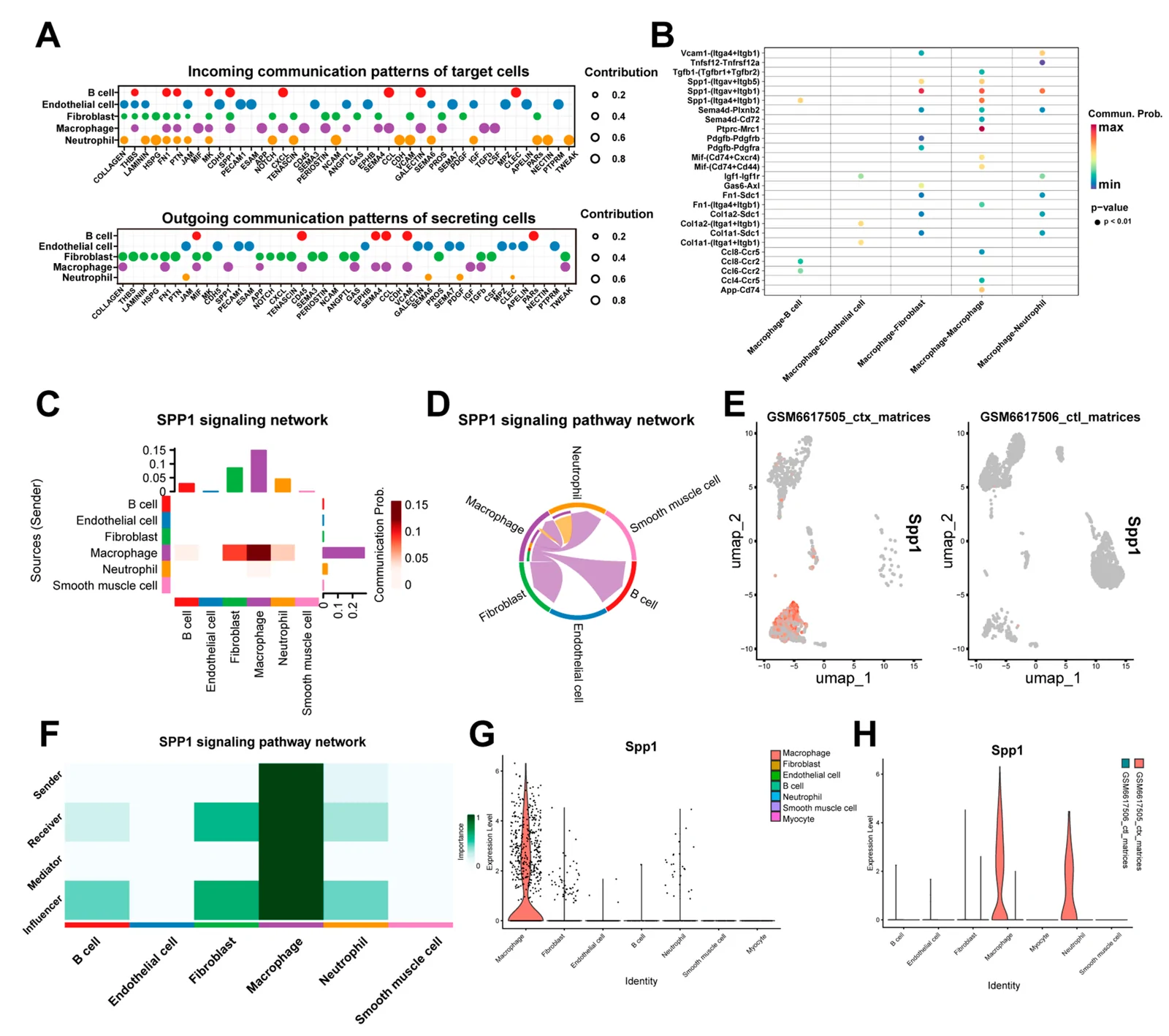
Ligand–receptor interaction analysis and signaling pathway identification. (**A**) Identification of the signaling pathways in the designated receptor and ligand cells in the CTX-induced injury group. (**B**) Analysis of interactions between macrophages and other cells. (**C**,**D**) Visualization of the selected *SPP1* signaling pathway. (**E**,**G**,**H**) Expression levels of *SPP1* in different cell types of the samples. (**F**) Calculation and visualization of *SPP1* Network centrality scores.

**Figure 3:**
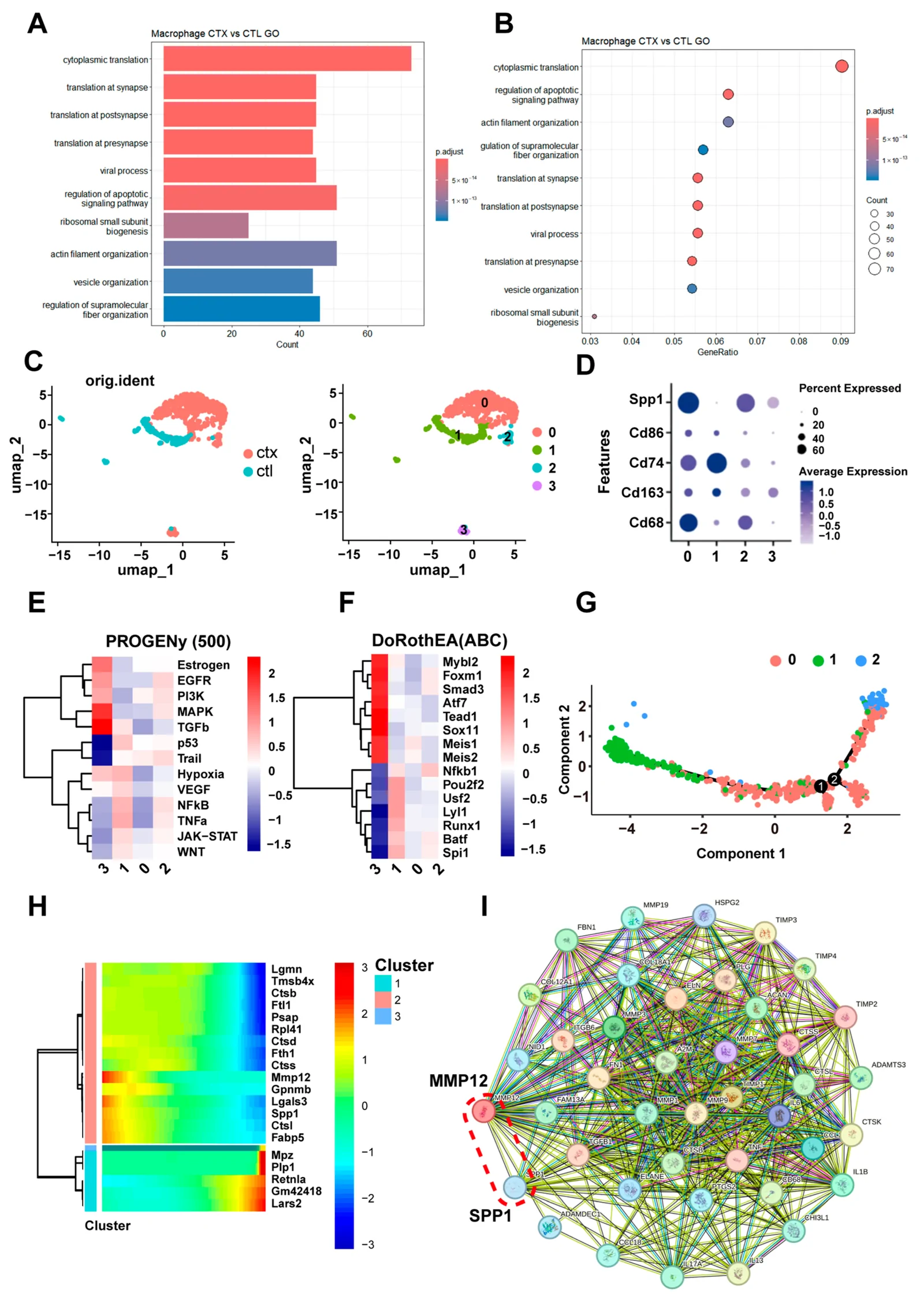
CTX-induced microenvironmental remodeling and the pseudotime trajectory of *MMP12/SPP1*-positive macrophage subset with an M4-like transcriptional signature. (**A**,**B**) GO enrichment analysis of macrophages in the CTX group and Control group. (**C**) Dimensionality reduction in macrophage re-clustering. (**D**) Re-annotation of the macrophages following re-clustering. (**E**) Estimation of pathway activity in macrophage immune cells using PROGENy. (**F**) Activity analysis of transcription factors using DoRothEA. (**G**) Pseudotime analysis of macrophages. (**H**) Heatmap of dynamically expressed genes along pseudotime in macrophages. (**I**) STRING derived protein–protein association network, with MMP12-SPP1 connection highlighted and enlarged in the inset.

To define the cellular landscape of skeletal muscle after acute injury, we performed scRNA-seq on cardiotoxin (CTX)-treated and control samples. After quality control and batch correction, unsupervised Seurat clustering identified eight cell clusters, corresponding to seven major lineages based on canonical markers: B cells, endothelial cells, fibroblasts, macrophages, myocytes, neutrophils, and smooth muscle cells (Figure 1A–D). CellChat analysis predicted macrophages as a major communication hub in the injured microenvironment, with high predicted connectivity and outgoing/incoming signaling activity (Figure 1E,F). Outgoing signaling pattern analysis further suggested that macrophages, fibroblasts, and B cells share similar communication profiles (Figure 1G,H and Figure 2A). Ligand–receptor mapping identified *SPP1* signaling as a central macrophage-driven pathway (Figure 2B,D), with high expression of both *SPP1* and its receptors predominantly in macrophages (Figure 2E,G,H). Network centrality analysis further indicated that macrophages act as both major senders and receivers (autocrine signaling), while fibroblasts are key secondary targets (paracrine signaling) (Figure 2F), suggesting that the *SPP1* axis is a major route by which macrophages regulate local fibrosis. Differential expression analysis of CTX macrophages showed enrichment of pathways related to protein synthesis, ribosome biogenesis, and cytoskeletal remodeling (Figure 3A,B). Subclustering identified four macrophage subsets (Clusters 0–3), among which Clusters 0, 2, and 3 were expanded after injury and displayed strong *SPP1* expression (Figure 3C,D). PROGENy analysis indicated increased TNFα, TGFβ, and PI3K pathway activity in injury-associated macrophages, while DoRothEA analysis showed activation of remodeling-associated transcription factors (e.g., Foxm1, Smad3, Sox11) and suppression of homeostatic regulators (e.g., Spi1, NF-κB) (Figure 3E,F). Pseudotime analysis further revealed a progressive transition of macrophages toward an injury-associated terminal state (Figure 3G), accompanied by marked upregulation of *MMP12*. Notably, *SPP1* expression positively correlated with *MMP12* and increased in parallel along the trajectory (Figure 3H), defining an *MMP12-SPP1* molecular program characteristic of M4-like macrophages that likely drives profibrotic remodeling during skeletal muscle repair.

Further, we established a protein–protein interaction (PPI) network to analyze relationship between *MMP12* and *SPP1* using STRING. The result suggested a strong functional association (Figure 3I). Previous studies have identified CXCL4-induced M4-like macrophages as a pro-fibrotic macrophage state associated with fibrotic diseases, characterized by high expression of *MMP12*, *SPP1*, *MMP7*, and *S100A8*. We therefore asked whether this injury-associated *MMP12/SPP1*-positive macrophage subset shares transcriptional features with CXCL4-induced M4-like macrophages. To this end, we compared each macrophage subcluster with the publicly available CXCL4-induced M4-like macrophage transcriptome (GSE20484). Human genes were mapped to mouse orthologs, and M4-like signature scores were calculated for each macrophage subcluster. A subcluster was considered to have an M4-like transcriptional signature if it showed: (1) enrichment of the CXCL4-induced M4-like signature from GSE20484; (2) high co-expression of *MMP12* and *SPP1*; (3) injury-associated expansion in the CTX-injured muscle; and (4) localization along the injury-associated pseudotime trajectory. Among the macrophage subclusters, sub0 and sub1 showed relatively high M4-like signature scores. Sub0 was predominantly derived from the CTX-injured group and exhibited high co-expression of *MMP12* and *SPP1*. In contrast, sub1 was mainly derived from the control group and expressed much lower levels of *MMP12* and *SPP1*. Based on the criteria above, we define sub0 as the injury-expanded *MMP12/SPP1*-positve macrophage subset with an M4-like transcriptional signature (Figure S1).

### 2.2. CXCL4 Promotes Macrophage Polarization Toward an M4-like Phenotype

**Figure 4:**
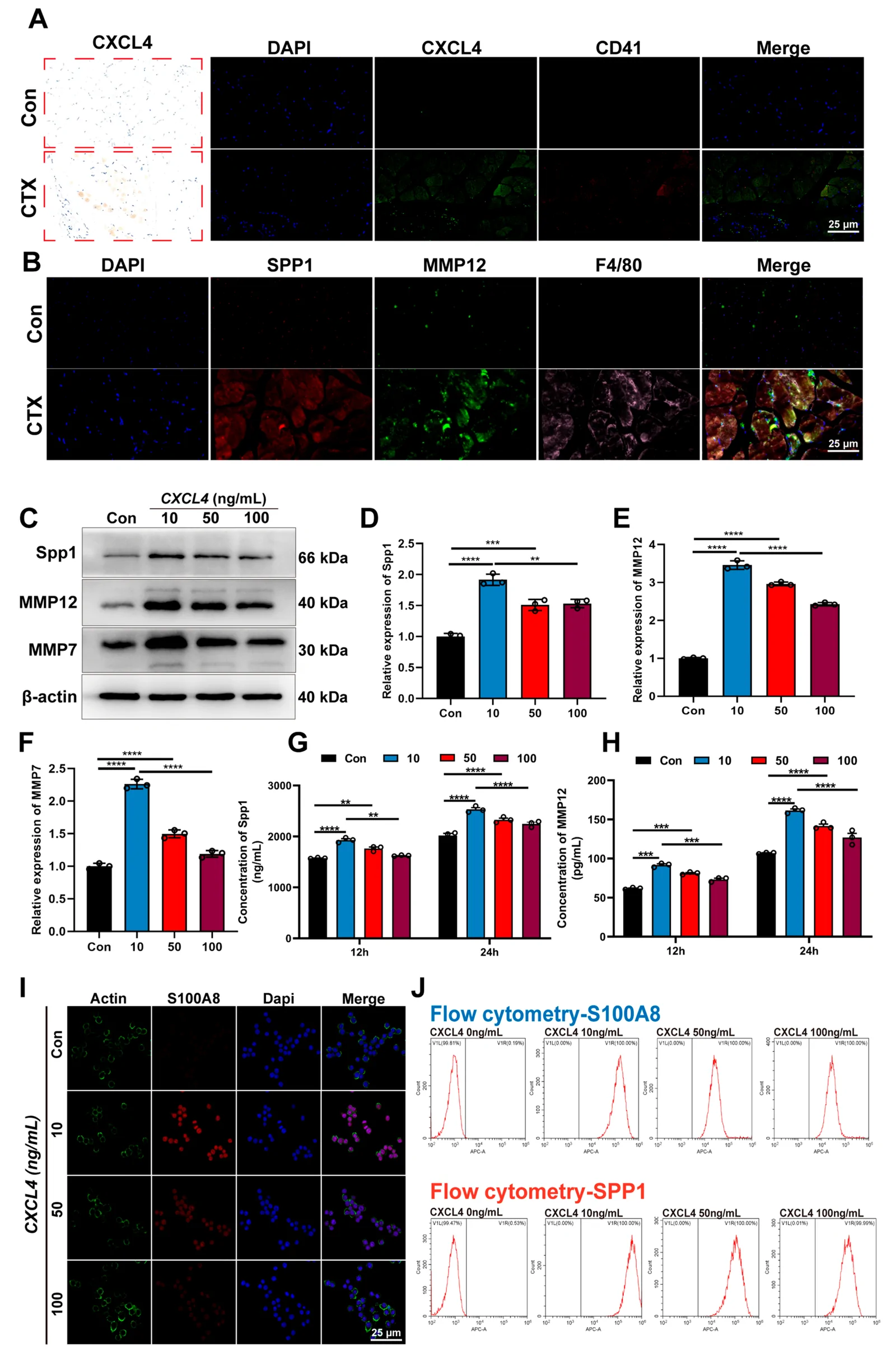
CXCL4 promotes macrophage polarization toward an M4-like phenotype. (**A**) Immunohistochemical and immunofluorescence staining of CXCL4 and CD41 in tibialis anterior (TA) muscle sections from control and CTX-injured BALB/c mice. Scale bar: 25 μm. (**B**) Representative multiplex immunofluorescence images of F4/80, SPP1, and MMP12 in TA muscle sections from control and CTX-injured mice. Scale bar: 25 μm. (**C**–**F**) Western blot analysis of SPP1, MMP12, and MMP7 expression in RAW264.7 macrophages treated with recombinant CXCL4 at the indicated concentrations for 24 h. (**G**,**H**) ELISA quantification of SPP1 and MMP12 concentrations in the culture supernatant of RAW264.7 macrophages treated with CXCL4 at the indicated concentrations for 12 h and 24 h (*n* = 3). (**I**) Representative immunofluorescence images of S100A8 in RAW264.7 macrophages under different CXCL4 treatments. Scale bar: 25 μm. (**J**) Flow cytometry quantification of the percentage of S100A8-positive macrophages after CXCL4 treatment at the indicated concentrations. ** *p* < 0.01, *** *p* < 0.001, **** *p* < 0.0001.

Previous studies have established that CXCL4, a chemokine released by activated platelets, can induce macrophage polarization toward a pro-fibrotic M4-like phenotype characterized by elevated SPP1 and MMP12 expression [16,17]. We therefore examined whether CXCL4 is present in the injured muscle microenvironment and contributes to the generation of M4-like macrophages following acute skeletal muscle injury. Immunohistochemical staining revealed markedly elevated CXCL4 expression in CTX-injured muscle tissues compared with uninjured controls (Figure 4A). Immunofluorescence staining showed increased CD41 + platelet infiltration in the injured area, with co-localization of CXCL4 and CD41 signals, suggesting that platelets are an important source of CXCL4 (Figure 4A). These findings confirm that muscle injury triggers local CXCL4 upregulation associated with platelet accumulation. Multiplex immunofluorescence staining of TA muscle sections showed co-localization of F4/80, SPP1, and MMP12 in the injured area, consistent with the presence of an MMP12/SPP1-positive macrophage subset in injured muscle (Figure 4B).

To functionally validate the polarizing effect of CXCL4 on macrophages, we treated Raw264.7 cells with recombinant CXCL4 at 10, 50, and 100 ng/mL. Western blot analysis confirmed that low-dose CXCL4 (10 and 50 ng/mL) significantly upregulated the M4-like markers MMP7, MMP12, and SPP1, while high-dose CXCL4 (100 ng/mL) reduced their expression (Figure 4C–F). ELISA further verified increased extracellular levels of MMP12 and SPP1 under low-dose stimulation, indicating enhanced secretion (Figure 4G,H). Immunofluorescence analysis corroborated this trend, showing elevated S100A8 expression at 10 and 50 ng/mL and decreased expression at 100 ng/mL (Figure 4I). Flow cytometry also demonstrated an increase in the M4-like macrophage population at 10 and 50 ng/mL, with a relative reduction at 100 ng/mL (Figure 4J). Collectively, these results indicate that CXCL4 signal was increased in the injured muscle and showed spatially associated with infiltrating CD41 + platelets. In vitro, recombinant CXCL4 promoted an M4-like macrophage phenotype in RAW264.7 cells with maximal induction at 10 and 50 ng/mL and attenuated responses at 100 ng/mL.

### 2.3. M4-like Macrophages Promote Activation and Increase Viability of L929 Fibroblasts

**Figure 5:**
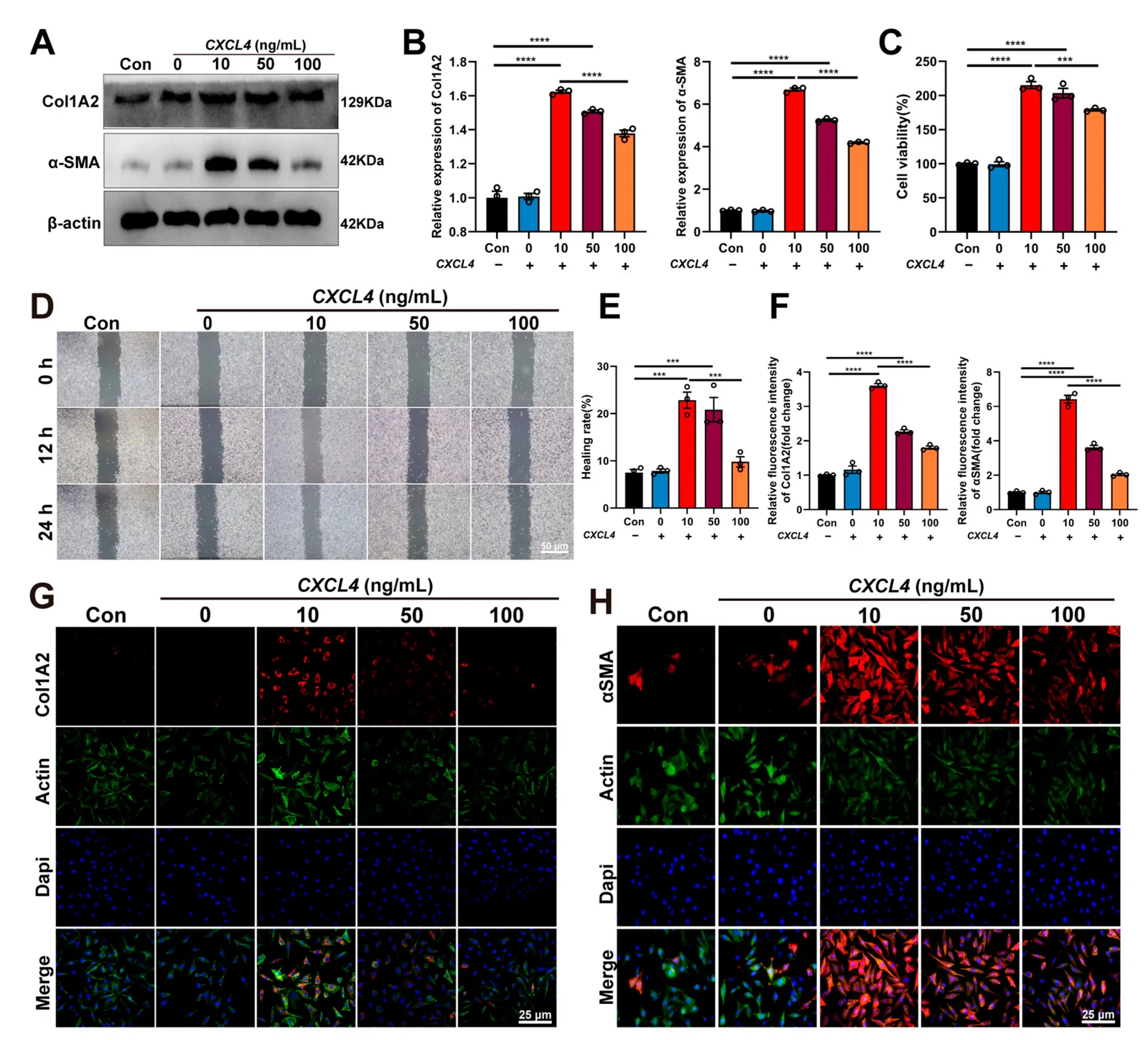
M4-like macrophages promote fibrogenic activation and viability of L929 fibroblasts. (**A**) The expression of αSMA and Col1A2 in L929 cells following co-culture with M4-like macrophages was detected by Western blot analysis. (**B**) Densitometric quantification of relative Col1A2 and α-SMA protein levels in L929 cells (*n* = 3). (**C**) Effect of M4-like macrophages on L929 cell viability was tested using CCK-8 assay. (**D**) Effect of M4-like macrophages on the migration of L929 cells. Representative images of wound healing assays were taken at 0, 12 h and 24 h after scratching. Scale bar: 250 μm. (**E**) Quantitative analysis of healing rate of L929 cells following co-culture with M4-like macrophages. (**F**) Quantitative analysis of relative fluorescence intensity of Col1A2 and α-SMA in L929 cells following co-culture with M4-like macrophages. (**G**) Representative fluorescence images of Col1A2 in L929 cells following co-culture with M4-like macrophages. Scale bar: 25 μm. (**H**) Representative fluorescence images of α-SMA in L929 cells following co-culture with M4-like macrophages. Scale bar: 25 μm. *** *p* < 0.001, **** *p* < 0.0001.

Based on established evidence linking M4-like macrophages to fibrotic pathology [18,19], we investigated whether M4-like macrophages exacerbate fibrosis during repair after acute skeletal muscle injury. We assessed fibroblast activation using a co-culture system in which L929 fibroblasts were incubated for 24 h with M4-like polarized macrophages (induced by 10, 50, or 100 ng/mL CXCL4). The CCK-8 assay revealed significantly enhanced viability in L929 fibroblasts co-cultured with M4-like macrophages, with the strongest effect at 10 ng/mL CXCL4 (Figure 5C). Western blot (Figure 5A,B) and immunofluorescence analyses (Figure 5F–H) confirmed that co-culture with M4-like macrophages significantly upregulated the expression of α-SMA and Collagen I in L929 cells, indicating differentiation into myofibroblasts. Similarly, the wound healing assay demonstrated accelerated migration of L929 cells under M4-like macrophages co-culture, also in a CXCL4 concentration-dependent manner (Figure 5D,E). The pro-fibrotic effect was dose-dependent, peaking at 10 ng/mL CXCL4. In summary, M4-like macrophages activate L929 fibroblasts by promoting myofibroblast transition, proliferation, and migration in a CXCL4 concentration-dependent manner.

### 2.4. M4-like Macrophages Impair Myogenesis by Suppressing C2C12 Cell Viability and Differentiation

**Figure 6:**
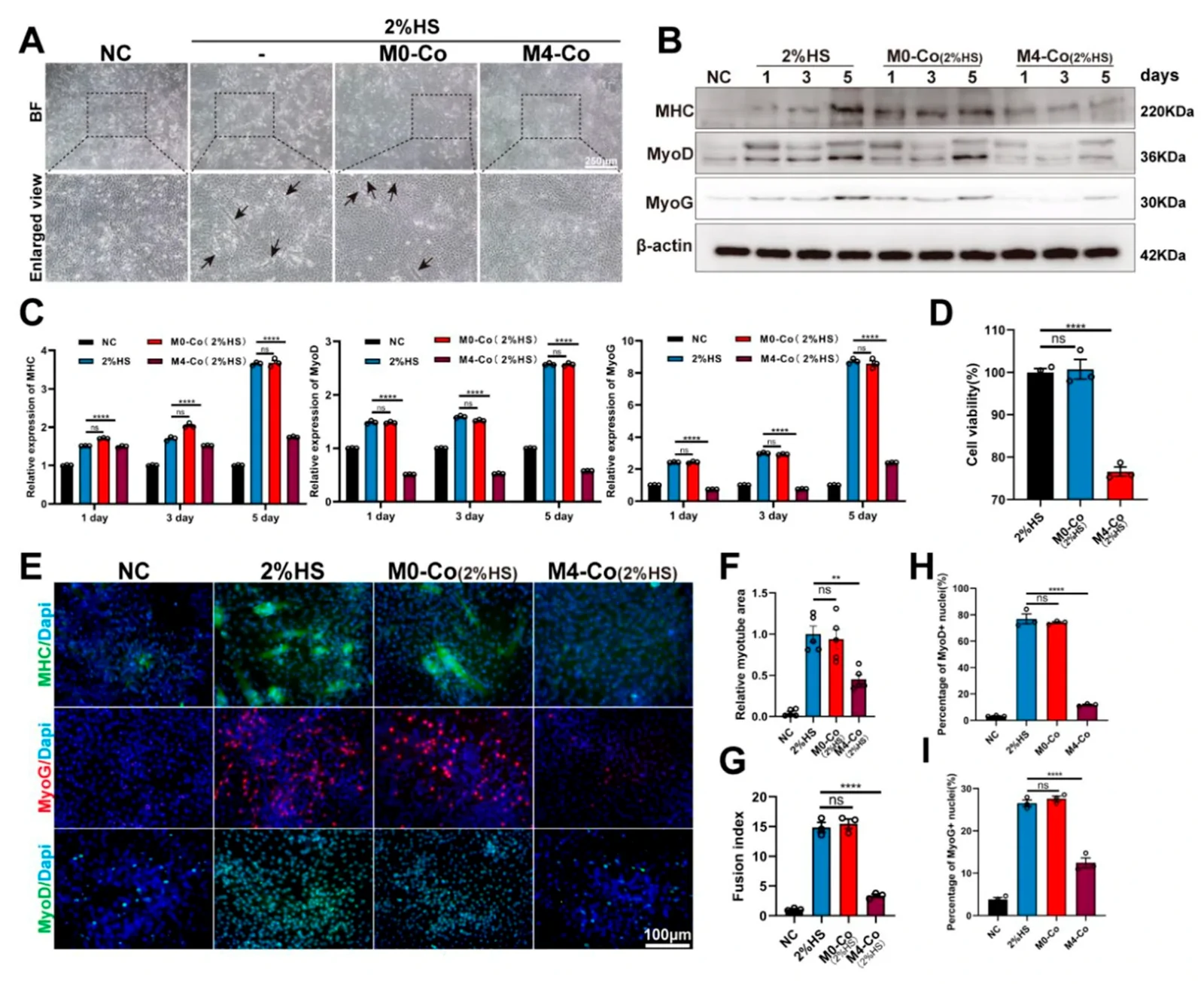
M4-like macrophages impair myogenesis by suppressing C2C12 cell viability and differentiation. (**A**) Representative images of C2C12 cells differentiation after different treatment. Black arrows indicate differentiated myotubes. Scale bar: 250 μm. (**B**) The expression of MHC, MyoD and MyoG of C2C12 cells was detected by Western blot analysis. (**C**) Densitometric quantification of relative MHC, MyoD, and MyoG protein levels by Western blot (*n* = 3). (**D**) Effect of M4-like macrophages on C2C12 cell viability was tested using CCK-8 assay. (**E**) Representative fluorescence images of MHC, MyoD and MyoG of C2C12 cells following co-culture with macrophages. Scale bar: 100 μm. (**F**,**G**) Quantitative analysis of fusion index (*n* = 3) and relative myotube area of C2C12 cells following co-culture (*n* = 5). The fusion index was calculated as the percentage of nuclei within multinucleated (≥2 nuclei) myotubes relative to all nuclei in MHC-positive cells. Relative myotube area was calculated as the ratio of MHC-positive area to the total field area and normalized to the control group (*n* = 3 for fusion index; *n* = 3 for relative myotube area). (**H**,**I**) Quantitative analysis of percentage of MyoD-positive(H) and MyoG-positive(I) nuclei in C2C12 cells following different co-culture conditions (*n* = 3). Scale bar: 100 μm. ns, not significant, ** *p* < 0.01, **** *p* < 0.0001.

Upon muscle injury, quiescent satellite cells activate, proliferate, and differentiate into myoblasts, which fuse to form new muscle fibers. Macrophages critically regulate this process, exerting dual effects: they promote satellite cell proliferation and differentiation via trophic factors, but under pathological conditions, they may impede maturation and myogenic progression, impairing regeneration [20,21,22]. Given this critical interaction, we aimed to investigate whether M4-like macrophages influence myoblast responses using C2C12 myoblasts as an in vitro model. Morphological assessment by light microscopy revealed that co-culture with M4-like macrophages severely impaired C2C12 myoblast differentiation. In the control group, C2C12 cells efficiently fused into elongated, multinucleated myotubes aligned in parallel, whereas M4-like macrophages-co-cultured cells remained largely unfused with a mononuclear, spindle-shaped morphology, resulting in fewer and shorter myotubes (Figure 6A). Western blot analysis showed markedly reduced expression of the myogenic markers MyoD, MyoG, and MHC at day 1, 3, and 5 post induction, indicating suppression of the myogenic program (Figure 6B,C). The CCK-8 assay showed significantly reduced C2C12 cell viability following co-culture with M4-like macrophages, suggesting reduced proliferative capacity and compromised cell survival during early differentiation (Figure 6D). Immunofluorescence analyses confirmed stage-specific inhibition, with fewer MyoD^+^ and MyoG^+^ nuclei indicating impaired initiation and suppressed terminal differentiation. Additionally, diminished MHC staining demonstrated disrupted myotube formation, accompanied by decreased fusion index and reduced relative myotube area (Figure 6E–I). Collectively, these findings demonstrate that M4-like macrophages inhibit cell viability and differentiation of C2C12 myoblasts.

### 2.5. MMP12–SPP1 Interplay Mediates M4-like Macrophage Effects on L929 Fibroblasts and C2C12 Myoblasts

**Figure 7:**
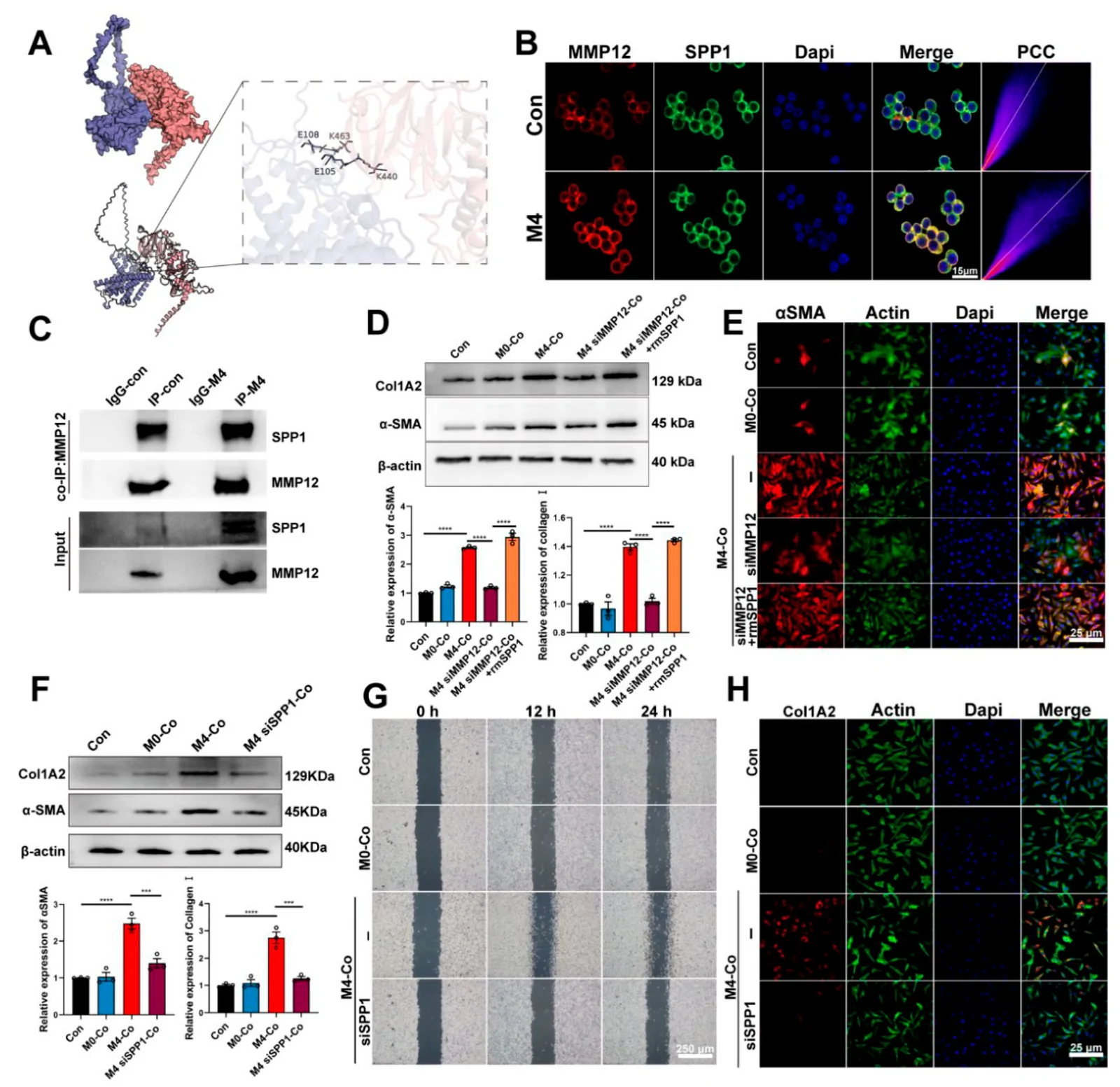
Functional association between MMP12 and SPP1 contributes to M4-like macrophage-mediated effects on L929 fibroblasts. (**A**) AlphaFold 3-generated structural model of the putative MMP12-SPP1 complex highlighting predicted interface residues. (**B**) Representative fluorescence images showing spatial colocalization of MMP12 and SPP1 in macrophages. Scale bar: 15 μm. (**C**) Representative co-immunoprecipitation analysis showing association between MMP12 and SPP1 in macrophages. (**D**) The expression of α-SMA and Col1A2 in L929 cells following co-culture with M4-like or M4-like transfected with si*MMP12* macrophages was detected by Western blot analysis. (**E**) Representative fluorescence images of α-SMA in L929 cells following co-culture with M4-like macrophages or M4-like macrophages transfected with si*MMP12*. (**F**) The expression of Col1A2 and α-SMA in L929 cells following co-culture with M4-like macrophages or M4-like macrophages transfected with si*SPP1*. (**G**) Effect of *SPP1*-knockdown M4-like macrophages or M4-like macrophages on the migration of L929 cells. Representative images of wound healing assays were taken at 0, 12 h and 24 h after scratching. Scale bar: 250 μm. (**H**) Representative fluorescence images of Col1A2 in L929 cells after co-culture with M4-like macrophages or M4-like macrophages transfected with si*SPP1*. Scale bar: 25 μm. *** *p* < 0.001, **** *p* < 0.0001.

**Figure 8:**
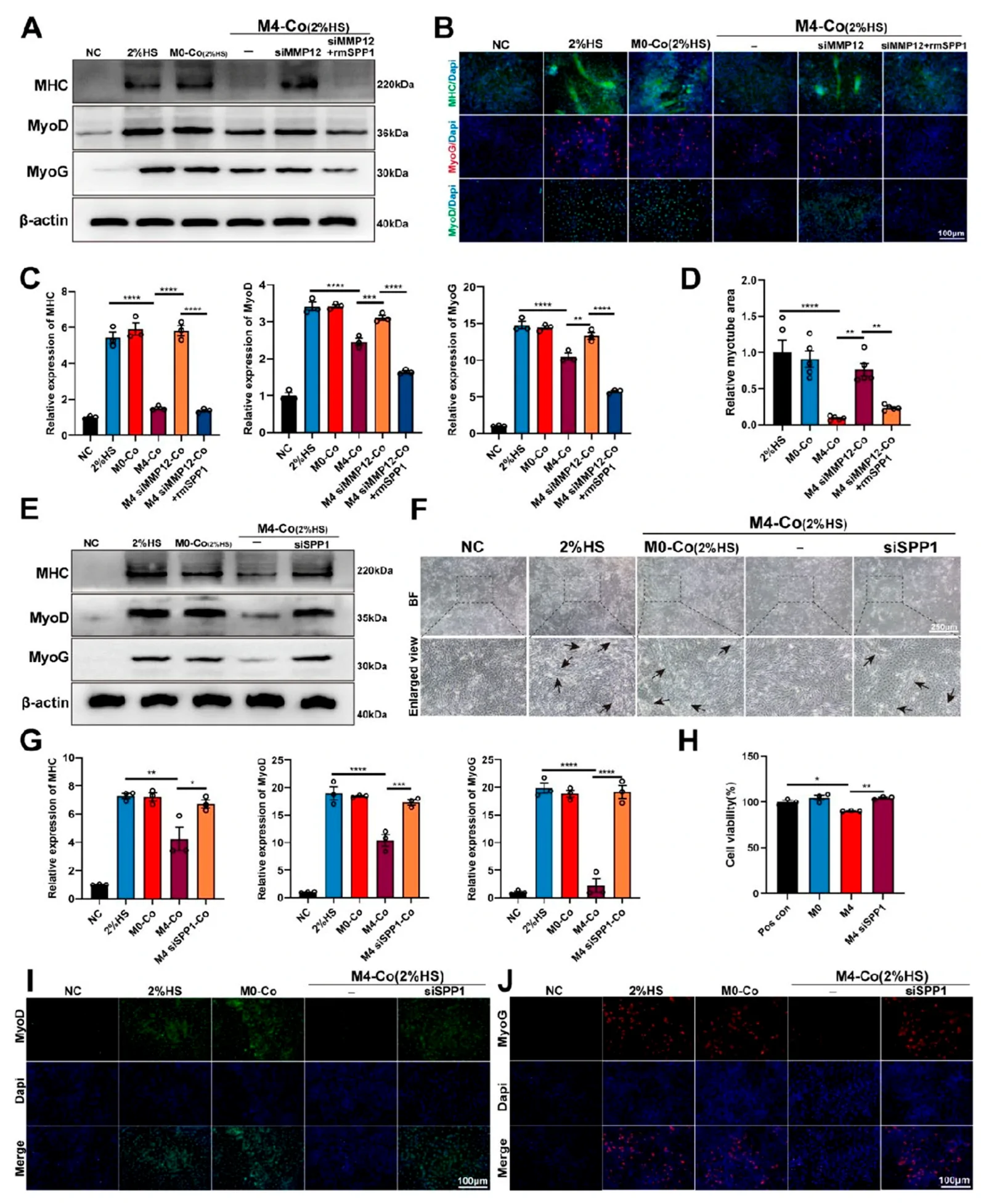
MMP12–SPP1 interplay mediates M4-like macrophage effects on C2C12 myoblasts. (**A**) The expression of MHC, MyoD and MyoG of C2C12 cells after co-culture with M4-like macrophages or M4-like macrophages transfected with si*MMP12* was detected by Western blot analysis. (**B**) Representative fluorescence images of MHC, MyoD and MyoG of C2C12 cells following co-culture with M4-like macrophages or M4-like macrophages transfected with si*MMP12*. Scale bar: 100 μm. (**C**) Densitometric quantification of relative MHC, MyoD, and MyoG protein levels by Western blot in C2C12 cells under different co-culture conditions (*n* = 3). (**D**) Quantitative analysis of relative myotube area of C2C12 cells under different co-cultures (*n* = 5). (**E**) The expression of MHC, MyoD and MyoG of C2C12 cells following co-culture with M4-like macrophages or M4-like macrophages transfected with si*SPP1* was detected by Western blot analysis. (**F**) Representative images of C2C12 cells differentiation after different co-cultures. Black arrows indicate differentiated myotubes. Scale bar: 250 μm. (**G**) Densitometric quantification of relative MHC, MyoD, and MyoG protein levels by Western blot in C2C12 cells under different co-culture conditions (*n* = 3). (**H**) Effect of M4-like macrophages and si*SPP1*-M4-like macrophages on C2C12 cells activity was tested using CCK-8 assay (*n* = 3). (**I**,**J**) Representative fluorescence images of MyoD and MyoG of C2C12 cells following co-culture. Scale bar: 100 μm. * *p* < 0.05, ** *p* < 0.01, *** *p* < 0.001, **** *p* < 0.0001.

Given that MMP12 has been reported to cleave SPP1 in other contexts, we hypothesized that the pro-fibrotic activity of M4-like macrophages dependent on the functional association between MMP12 and SPP1 [23,24,25,26]. To investigate this, we first explored MMP12-SPP1 interactions using AlphaFold3, which predicted a high-confidence interaction model. The model indicated a potential binding interface involving Lys463 and Lys440 of MMP12 and Glu105 and Glu108 of SPP1 (Figure 7A). Immunofluorescence further showed increased co-localization of MMP12 and SPP1 in M4-like macrophages compared with M0 macrophages (Figure 7B). The interaction predicted by AlphaFold3 was further supported by co-immunoprecipitation (co-IP), suggesting an association between the two proteins (Figure 7C). Consistent with their elevated protein levels in M4-like macrophage relative to M0 macrophages, this interaction was substantially enhanced under M4-like polarization conditions. To determine whether MMP12 mediates M4-like macrophage functions through interaction with SPP1, we knocked down *MMP12* in M4-like macrophages using siRNA and assessed the effects on fibroblasts and myoblasts. Western blot confirmed that the selected siRNA reduced MMP12 protein expression, and this siRNA was used for subsequent experiments (Figure S2A). In L929 fibroblasts co-cultured with *MMP12*-knockdown M4-like macrophages, Western blot and immunofluorescence analyses revealed significantly reduced expression of α-SMA and collagen I (Figure 7D,E and Figure S2C,D). The CCK-8 assay showed suppressed cell viability, and the wound healing assay demonstrated impaired migratory capacity. Notably, these inhibitory effects were largely reversed upon addition of recombinant SPP1 (Figure S2B,E). In C2C12 myoblasts co-cultured with *MMP12*-knockdown M4-like macrophages, Western blot and immunofluorescence analyses showed restored expression of MyoD, MyoG, and MHC, accompanied by increased myogenic differentiation and myotube formation (Figure 8A–D and Figure S4A,D,E). Thus, our findings indicate that M4-like macrophages exert a dual role in promoting fibroblast activation while suppressing myoblast differentiation through MMP12-SPP1 interaction.

### 2.6. SPP1 Mediates M4-like Macrophage-Driven Fibrogenic Activation and Myogenic Suppression

To investigate whether M4-like macrophages regulate fibrosis and myogenesis through SPP1 secretion, we knocked down *SPP1* in M4-like macrophages using siRNA. Western blot confirmed that the two selected siRNA sequences reduced SPP1 protein expression, and these sequences were used for subsequent experiments (Figure S3A). *SPP1* knockdown significantly attenuated the pro-fibrotic effect of M4-like macrophages on L929 fibroblasts. Western blot (Figure 7F and Figure S3C) and immunofluorescence analyses (Figure 7H and Figure S3D) revealed that co-culture with M4-like macrophages markedly upregulated α-SMA and collagen I expression in L929 cells, whereas this effect was substantially diminished upon *SPP1* knockdown. Furthermore, the CCK-8 and wound healing assays showed that *SPP1* knockdown reduced M4-like macrophage-induced L929 cell viability and migratory capacity (Figure 7G and Figure S3B). Conversely, the inhibitory effect of M4-like macrophages on C2C12 myoblasts was markedly reversed following *SPP1* knockdown. The CCK-8 assay further confirmed enhanced C2C12 cell viability upon *SPP1* knockdown (Figure 8H). Western blot (Figure 8E,G) and immunofluorescence analyses (Figure 8I,J and Figure S4C) showed that *SPP1* knockdown restored MyoD, MyoG, and MHC expression, accompanied by increased myogenic differentiation and myotube formation (Figure 8F). Taken together, these findings suggest that M4-like macrophages mediate fibroblast activation and impair myoblast differentiation through SPP1 secretion.

### 2.7. Transcriptome Sequencing and Bioinformatics Analysis

**Figure 9:**
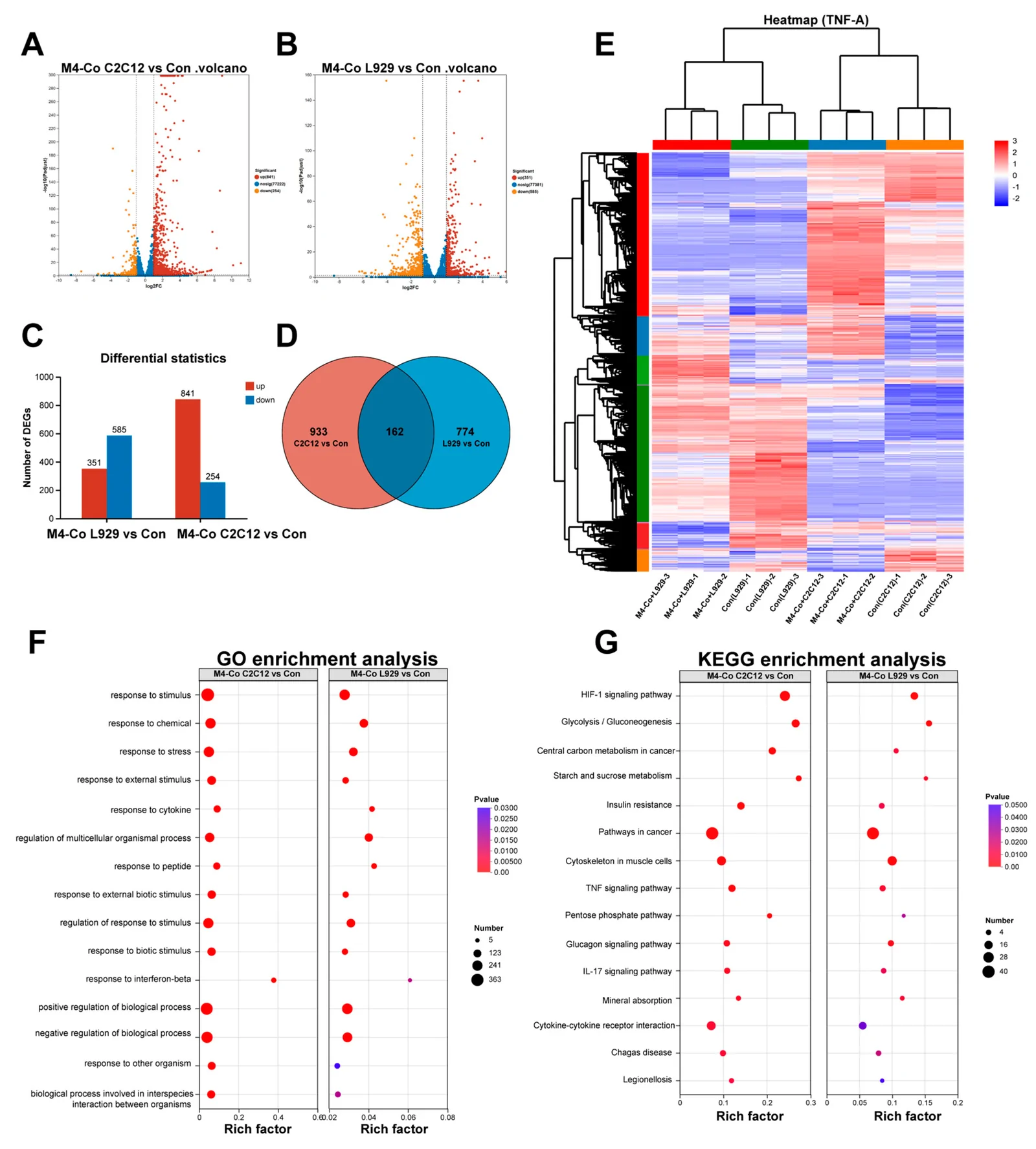
Transcriptome sequencing and bioinformatics analysis. (**A**–**C**) Analysis of differential genes of L929 and C2C12 cells following co-culture with M4 macrophages. (**D**) Venn diagram of the differential genes expressed by L929 and C2C12 cells. (**E**) Heat map analysis of differential genes of L929 and C2C12 cells following co-culture with M4 macrophages. (**F**) GO enrichment analysis of L929 and C2C12 cells following co-culture with M4 macrophages. (**G**) KEGG enrichment analysis of L929 and C2C12 cells following co-culture with M4 macrophages.

To further elucidate the mechanism underlying the opposing effects of M4-like macrophages on fibroblasts and myoblasts, we performed transcriptomic analysis on L929 fibroblasts and C2C12 myoblasts following co-culture with M4-like macrophages (Figures S5 and S6). Transcriptome sequencing revealed that co-culture with M4-like macrophages resulted in 351 upregulated and 585 downregulated genes in L929 fibroblasts, while C2C12 myoblasts exhibited 841 upregulated and 254 downregulated genes, with 162 differentially expressed genes overlapping between the two cell types (Figure 9A–D). Notably, RNA-seq analysis revealed that both cell types shared a common transcriptional trend characterized by the activation of inflammatory pathways. GO analysis demonstrated similar expression patterns in both cell types, with “response to cytokine” being significantly enriched (Figure 9F). KEGG pathway analysis further revealed that the TNF-α signaling pathway was markedly activated in both L929 and C2C12 cells (Figure 9E,G).

### 2.8. M4-like Macrophage Activates the NF-κB Pathway Through Autocrine SPP1

**Figure 10:**
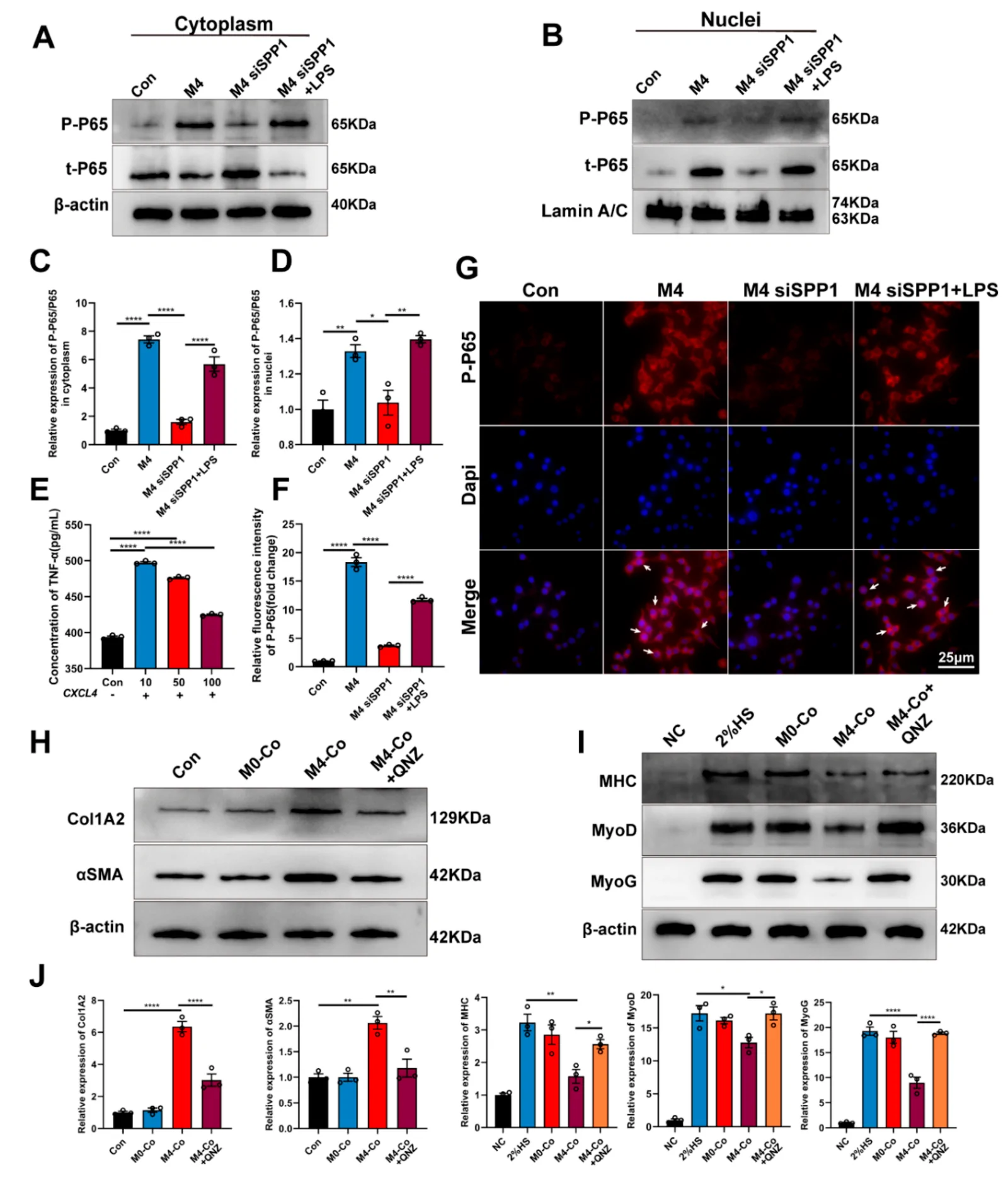
M4-like macrophages activate the NF-KB pathway through autocrine SPP1. (**A**) The expression of P-P65 and total P-65 (t-P65) in the cytoplasm of macrophages was detected by Western blot analysis. (**B**) The expression of P-P65 and total P-65 (t-P65) in the nuclei of macrophages was detected by Western blot analysis. (**C**) Densitometric quantification of relative P-P65 and t-P65 protein levels by Western blot in the cytoplasmic fraction of macrophages (*n* = 3). (**D**) Densitometric quantification of relative P-P65 and t-P65 protein levels by Western blot in the nuclear fraction of macrophages (*n* = 3). (**E**) Quantitative analysis of the concentration of TNF-α in the supernatant (*n* = 3). (**F**) Quantitative analysis of the relative fluorescence intensity of P-P65 in different group. (**G**) Representative fluorescence images of P-P65 in the macrophages showing the translocation of P-P65. Scale bar: 25 μm. Arrows indicate cells showing nuclear translocation of phosphorylated P65 (P-P65). (**H**) The expression of Col1A2 and α-SMA in L929 cells following different co-cultures was detected by Western blot analysis. (**I**) The expression of MHC, MyoD and MyoG in C2C12 cells following different co-cultures was detected by Western blot analysis. (**J**) Densitometric quantification of relative Col1A1, α-SMA, MHC, MyoD, and MyoG protein levels by Western blot (*n* = 3). * *p* < 0.05, ** *p* < 0.01, **** *p* < 0.0001.

Based on our finding that M4-like macrophages regulate fibroblasts and myoblasts via MMP12-SPP1 interplay, we next investigated the downstream signaling pathways involved. Transcriptome sequencing of L929 and C2C12 cells co-cultured with M4-like macrophages revealed activation of the TNF-α signaling pathway in both cell types. Prior studies have shown that SPP1 can activate NF-κB signaling in macrophages and promote TNF-α production [27,28,29]. Given that SPP1 can act in an autocrine manner, we hypothesized that M4-like macrophages utilize an autocrine SPP1 loop to activate NF-κB and enhance their own TNF-α secretion, which then mediates the opposing effects on fibroblasts and myoblasts. To test this, we examined NF-κB pathway activation in M4-like macrophages. Western blot analysis showed significantly increased phospho-P65 and phospho-IκB levels in M4-like macrophages compared with controls, indicating NF-κB activation. This effect was markedly suppressed by *SPP1* knockdown and restored by an NF-κB agonist (Figure 10A–D). ELISA demonstrated increased secretion of TNF-α by M4-like macrophages (Figure 10E), and immunofluorescence confirmed enhanced nuclear translocation of P-P65 (Figure 10F,G). These results indicate that SPP1 activates NF-κB signaling and increases TNF-α secretion in M4-like macrophages. We next asked whether NF-κB signaling contributes to the effects on target cells. Co-culture with the NF-κB inhibitor QNZ significantly reduced α-SMA and collagen I expression in L929 fibroblasts while restoring MyoD, MyoG, and MHC expression in C2C12 myoblasts (Figure 10H–J). However, QNZ is a pharmacological inhibitor that may affect multiple cell types in the co-culture system. Therefore, these data suggest that M4-like macrophages activate NF-κB signaling and increase TNF-α secretion via autocrine SPP1, and that TNF-α may contribute to fibroblast activation and suppression of myogenic differentiation.

### 2.9. Targeting Macrophage SPP1/NF-κB Signaling Attenuates Fibrosis and Improves Muscle Regeneration In Vivo

**Figure 11:**
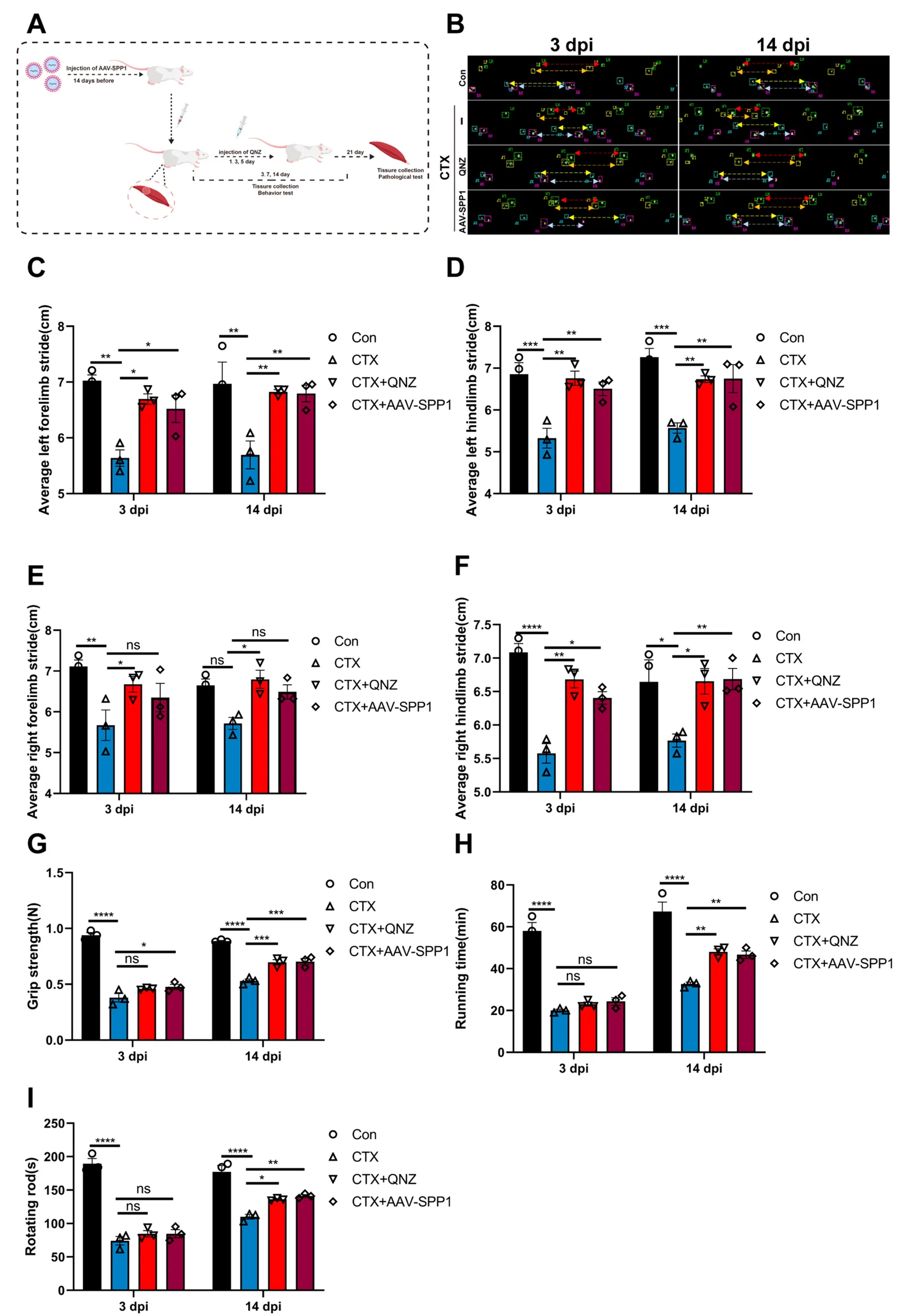
QNZ or AAV-Mediated Spp1 knockdown in macrophages improved functional recovery (**A**) The schematic overview of the in vivo experimental design. (**B**) The representative images of gait tracking via gait analysis. The length of arrows of the same color represents stride length. (**C**–**F**) Quantitative analysis of the stride length of mice in different groups at 3 and 14 days post-injuries (dpi). (**G**–**I**) Quantitative analysis of rotarod, grip strength and tread mill performance of mice in different groups. Data are presented as mean ± SEM. Each data point represents an individual mouse (*n* = 3 per group). Longitudinal data were analyzed using a mixed-effects model with animal as a random factor. Post hoc comparisons were performed within each time point (Day 3 and Day 14) using Tukey’s multiple comparisons test. * *p* < 0.05, ** *p* < 0.01, *** *p* < 0.001, **** *p* < 0.0001.

**Figure 12:**
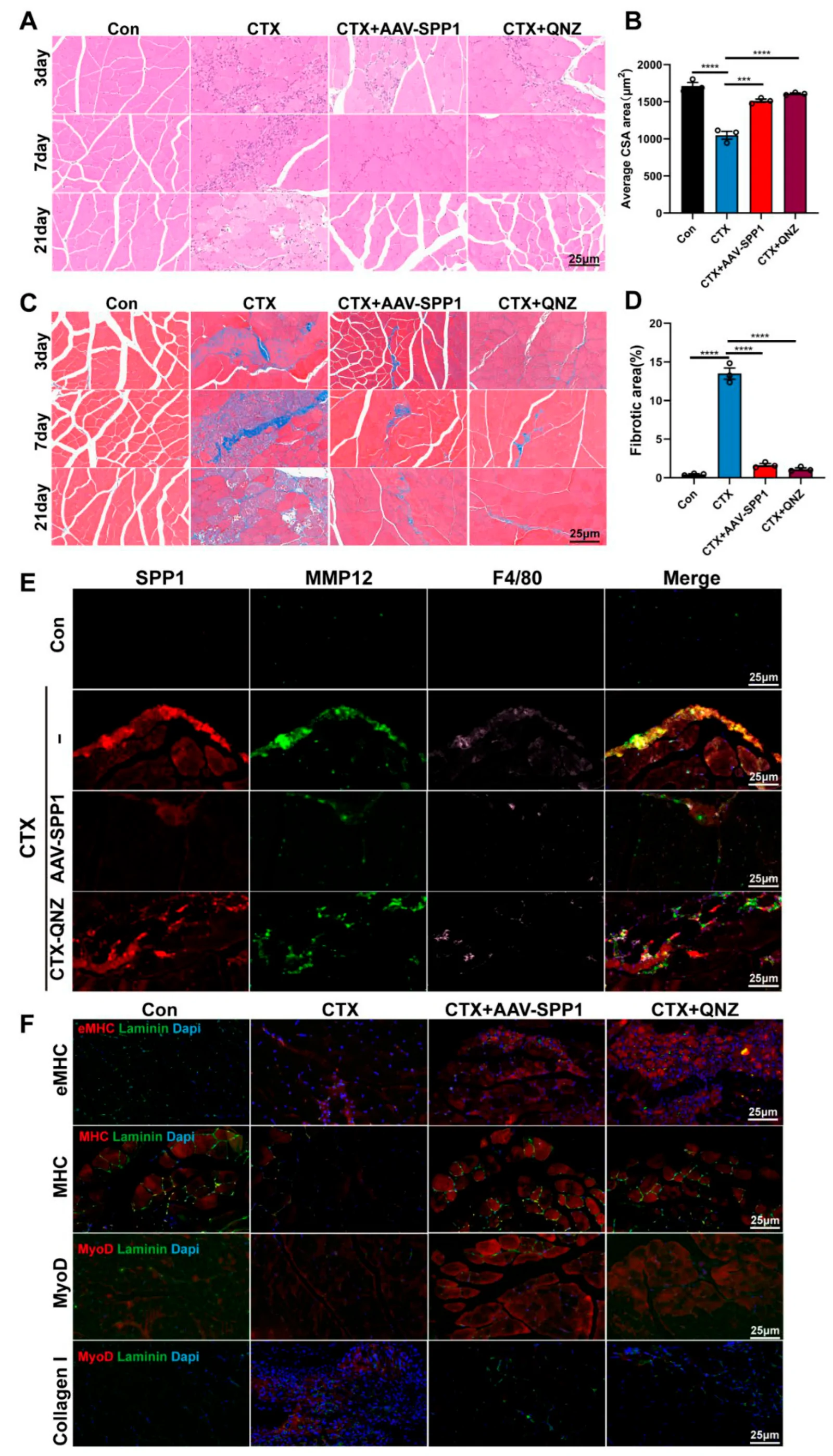
Histological analysis indicated improved recovery following QNZ treatment or AAV-Mediated Spp1 knockdown in macrophages. (**A**) H&E stain of the tibialis anterior (TA) in all experimental groups. (**B**) Quantitative analysis of the cross-sectional area (CSA) of the tibialis anterior (TA) in all experimental groups (*n* = 3). (**C**) The Masson stain of the tibialis anterior (TA) in all experimental groups (*n* = 3). (**D**) Quantitative analysis of the fibrotic area of the tibialis anterior (TA) in all experimental groups (*n* = 3). (**E**) The multiplex immunofluorescence staining of F4/80, SPP1, and MMP12 in the tibialis anterior (TA). Scale bar: 25 μm. (**F**) The immunofluorescence of eMHC, MHC, MyoD and Collagen I in the tibialis anterior (TA). Scale bar: 25 μm. *** *p* < 0.001, **** *p* < 0.0001.

Our in vitro findings demonstrate that M4-like macrophages promote fibrosis and inhibit myogenesis via the MMP12/SPP1/TNF-α axis. To determine whether this mechanism operates in vivo, we employed a mouse model of acute skeletal muscle injury induced by intramuscular injection of CTX. Mice were randomly assigned into four groups (Figure 11A): (1) a sham-operated control group, (2) a CTX injury model group, (3) a group receiving AAV-sh*SPP1*, and (4) a group treated with the NF-κB inhibitor (QNZ). Functionally, detailed gait analysis showed that both interventions restored stride length to near-normal levels by day 14, whereas the reduction persisted in the CTX model group (Figure 11B–F). Consistently, grip strength was similarly impaired across all injured groups at day 3, but recovery by day 14 was significantly greater in the AAV-sh*SPP1* and QNZ groups. Rotarod and treadmill tests further demonstrated markedly improved exercise endurance in both intervention groups, as evidenced by a longer latency to fall and increased running time. Together, these coordinated improvements in locomotion, strength, and endurance indicate robust functional recovery (Figure 11G–I). At day 3, 7, and 21 post-injury, histological analyses showed that CTX administration induced substantial collagen deposition by days 7 and 21, consistent with fibrotic remodeling. H&E staining at day 21 revealed impaired myofiber regeneration in the CTX group, characterized by persistent inflammatory cell infiltration, blurred myofiber boundaries, and intramyofiber vacuolization. Compared with the CTX group, the two intervention groups exhibited better muscle recovery outcomes, with improved skeletal muscle fiber morphology and larger cross-sectional area (CSA) (Figure 12A,B and Figure S7A). Masson’s trichrome staining confirmed reduced collagen accumulation in both intervention groups, accompanied by increased numbers of regenerating myofibers (Figure 12C,D and Figure S7B). Co-Immunofluorescence further demonstrated the presence of F4/80^+^MMP12^+^SPP1^+^ M4-like macrophages in injured muscle, with markedly reduced infiltration following *SPP1* knockdown (Figure 12E and Figure S7C). Collagen I staining was also significantly decreased in the intervention groups. Conversely, myogenic regeneration markers-including embryonic myosin heavy chain (eMHC), mature MHC, and the early transcription factor MyoD-were elevated relative to the CTX model group (Figure 12F). Collectively, these in vivo data indicate that targeting the MMP12/SPP1/TNF-α axis attenuates fibrosis, promotes muscle regeneration, and improves functional recovery after acute injury.

## 3. Discussion

The repair of acute skeletal muscle injury follows a finely tuned program of inflammation and remodeling, with macrophages serving as central regulatory hubs. Transcending the conventional M1/M2 dichotomy, emerging evidence reveals that high-dimensional macrophage subsets with unique functional identities populate the injured niche to fine-tune tissue restoration [30,31,32]. Here, we observed an *MMP12/SPP1*-positve macrophage subset with an M4-like transcriptional signature in acutely injured skeletal muscle. Previous studies have shown that CXCL4-induced M4-like macrophages exhibit impaired efferocytosis and contribute to pulmonary fibrosis in systemic sclerosis (SSc) [33], revealing that this state is associated with pathological tissue remodeling. In addition, HDAC9 has been reported to drive polarization toward an M4-like phenotype with elevated MMP12 expression, thereby accelerating plaque destabilization and atherosclerosis [34]. Consistent with these reports, we recapitulated CXCL4-driven M4-like polarization in vitro. Importantly, our in vitro data support an important role for this subset during acute muscle repair, as it promotes fibroblast activation and myofibroblast differentiation while restraining myogenic progression.

M4-like macrophages are characterized by high expression of *MMP12* and *SPP1*, suggesting that this molecular signature may contribute to remodeling of the muscle repair microenvironment. MMP12 (macrophage metalloelastase) is a macrophage-enriched matrix metalloproteinase that is secreted into the extracellular space [35,36]. Emerging evidence identifies MMP12 as a key driver of pathological fibrosis. In the context of silicosis, for instance, macrophage-derived MMP12 has been shown to sequester within the extracellular matrix, where it sustains endothelial injury throughout both the inflammatory and fibrotic phases [37]. Similarly, macrophage-derived MMP12 promotes subretinal fibrosis [38]. On the basis of these observations, we posited that M4-like macrophages may exert their regulatory effects through MMP12. Consistent with this hypothesis, siRNA-mediated *MMP12* silencing in our co-culture system promoted myogenic progression, as reflected by enhanced myotube fusion, while attenuating fibroblast activation and myofibroblast differentiation. Together, these findings support a functional role for MMP12 in mediating the pro-fibrotic and anti-myogenic effects of M4-like macrophages during muscle repair.

Although MMP12 and SPP1 have been reported to express coordinately in tumors and myocardial inflammation, the mechanisms in skeletal muscle pathology remain unclear [39,40]. Here, we provide evidence for a functional interaction between MMP12 and SPP1 within the injured muscle microenvironment. SPP1 (osteopontin, OPN) is a multifunctional extracellular matrix glycoprotein and cytokine that mediates cell–matrix interactions through RGD- and non-RGD-dependent domains. It is induced during inflammation and tissue repair and is constitutively expressed in tissues such as bone and kidney tissues [41,42]. Notably, *SPP1*^+^ macrophages have been shown to promote coronary perivascular adipose tissue (PVAT) fibrosis through SPP1-CD44 integrin signaling, with this pathway correlating with stenosis severity [43]. Building on these insights, we hypothesized that SPP1 functions as a key downstream effector of MMP12 in shaping skeletal muscle repair outcomes. Indeed, the beneficial effects of *MMP12* knockdown on myoblast differentiation and fibroblast activation were largely reversed by recombinant SPP1 supplementation. Moreover, *SPP1* knockdown mitigated the deleterious effects of M4-like macrophages on myogenesis and fibroblast-to-myofibroblast transition.

These findings further support a functional association between MMP12 and SPP1 in this process. SPP1 acts in an autocrine manner to activate NF-κB signaling in M4-like macrophages and increase TNF-α secretion. Secreted TNF-α may contribute to fibroblast activation and fibrosis while inhibiting myoblast differentiation. Consistent with our observations, TNF-α has been widely reported to induce myotube atrophy and impair myotube maturation [44,45,46]. Intriguingly, the effects of TNF-α on L929 fibroblasts appear to be more complex. Although many studies have reported inhibitory effects, others have shown that TNF-α can promote L929 fibroblast activation and proliferation [47,48,49]. This apparent discrepancy suggests that TNF-α exerts dual, context-dependent effects on fibroblasts. The outcome is likely determined by factors such as concentration, duration of exposure, and the presence of co-stimulatory signals within the local microenvironment.

In vivo, *SPP1* knockdown or NF-κB pathway inhibition ameliorated fibrosis, enhanced myogenic regeneration, and restored muscle function, supporting the therapeutic relevance of this pathway. These convergent outcomes support a role for MMP12/SPP1/NF-κB signaling, with TNF-α as potential downstream mediator and suggest that pathway-directed intervention may provide a more precise therapeutic strategy. Nevertheless, several limitations should be acknowledged. First, this study is restricted to acute CTX-induced injury, and whether this axis operates similarly in chronic muscle disease settings, including muscular dystrophy, remains to be determined. Second, although MMP12 has been reported to cleave SPP1 in other systems, direct proteolytic processing of SPP1 by MMP12 was not demonstrated in this study. Further biochemical studies, such as in vitro cleavage assays, mass spectrometry identification of cleavage sites, and cleavage-site mutants are required to test whether MMP12 directly processes SPP1. In addition, the contribution of downstream effectors beyond TNF-α remains incompletely defined. Another important limitation is the pooling design of the GSE214892 dataset. Thus, inter-animal variability and animal-level statistical comparisons cannot be assessed. The scRNA-seq should therefore be considered descriptive and hypothesis-generating, while the functional conclusions rely on independently replicated in vitro and in vivo experiments. Finally, the receptor-mediated mechanisms underlying the divergent effects of TNF-α on fibroblasts and myoblasts require further mechanistic investigation.

## 4. Materials and Methods

### 4.1. Regents and Antibodies

C2C12 cell line, L929 cell, and RAW 264.7 cell were all purchased from Procell Life Science & Technology (Wuhan, Hubei, China). C2C12 cells and RAW 264.7 cells were cultured in DMEM medium while L929 cells were cultured in MEM medium (Gibco, Thermo Fisher Scientific, Waltham, MA, USA). All medium contained 10% fetal bovine serum (FBS; Procell Life Science & Technology, Wuhan, China) and 1% penicillin-streptomycin (P/S; Procell Life Science & Technology, Wuhan, China). Cells were cultured in a humidified incubator at 37 °C with 5% CO_2_. For co-culture experiments, RAW264.7 cells were first stimulated with CXCL4 for 24 h in the upper chambers of transwell inserts. These cells were then co-cultured with either C2C12 cells, which were differentiated in medium containing 2% horse serum, or with L929 cells cultured under standard conditions. To assess the activation of L929 fibroblasts, the primary antibody for α-smooth muscle actin (α-SMA) was purchased from Zenbio (Chendu, China, Cat#R23450), and the primary antibody for collagen I (Collagen I) was purchased from Zenbio (Chendu, China, Cat#26615). The primary antibody for β-actin was purchased from Affinity (Liyang, China,Cat#AF7018). For the validation of M4-like macrophage polarization, the primary antibody for MMP12 was purchased from Huabio (Hangzhou, China, Cat#ET1602-42), while the primary antibodies for SPP1, S100A8, and MMP7 were purchased from Proteintech (Wuhan, China, Cat#22952-1-AP, Cat#15792-1-AP, Cat#10374-2-AP), the secreted level of SPP1 and MMP12 was detected using ELISA kit from Finetest (Wuhan, China, Cat#EM0151, Cat#EM0141). To investigate the colocalization of MMP12 and SPP1, a multiplex fluorescence labeling kit was purchased from Zenbio (Chendu, China, Cat#18002FC). To evaluate the effects of M4-like macrophages on C2C12 myoblasts, the primary antibody for myosin heavy chain (MHC) was purchased from Proteintech (Wuhan, China, Cat#22281-1-AP), the primary antibodies for myogenic differentiation (MyoD), embryonic myosin heavy chain (eMHC)were purchased from Proteintech (Wuhan, China, Cat#18943-1-AP, Cat#22287-1-AP), and myogenin (MyoG) were purchased from Zenbio (Chendu, China, Cat#R382257). To explore the involvement of the NF-κB signaling pathway in M4-like macrophages, the primary antibodies for P65, phospho-P65 (P-P65), IκB, and phospho-IκB (P-IκB) were purchased from Zenbio (Chendu, China, Cat#380172, Cat#310013, Cat#R380682, Cat#340776). LPS chosen as the agonist of NF-κB signaling pathway was purchased from Biosharp (Hefei, China, Cat#BS904). QNZ was purchased from Selleck (Shanghai, China, Cat#S4902). The macrophage-specific AAV vector harboring shRNA targeting *SPP1* was constructed by GenePharma Corporation (Shanghai, China). Recombinant CXCL4 was purchased from MedChemExpress (Monmouth Junction, NJ, USA). The CCK8-kit was purchased from APExBIO (Houston, TX, USA).

### 4.2. Animal Treatment

Male BALB/c mice aged 4 weeks were purchased from GemPharmatech Co., Ltd (Nanjing, China). To induce muscle injury, 50 µL of 10 µM cardiotoxins (CTX; MCE, Monmouth Junction, NJ, USA) was injected into the left tibialis anterior (TA) muscle [10,11] according to the guidelines and relevant laws by the Ethics Committee for the Second affiliated hospital of Chongqing Medical University (IACUC-SAHCQMU-2025-0256). The animals were randomly divided into four groups: (1) Control group (untreated, intact mice), (2) CTX injury group, (3) CTX + AAV-sh*SPP1* group, and (4) CTX + QNZ group. AAV8 expressing shRNA targeting *Spp1* was injected intramuscularly into the left tibialis anterior muscle at a concentration of 2 × 10^12^ vg/mL in a volume of 5 uL, corresponding to a total dose of 1 × 10^10^ vg per TA muscle. The AAV-scramble control (AAV8, same dose and volume) was injected in the same manner. In the CTX + QNZ group, mice received intraperitoneal injections of QNZ (a NF-κB pathway inhibitor) on 1, 3, and 5 days post-CTX injury to inhibit TNF-α expression. Functional recovery was assessed at 3, 7, and 21 days post-injury using treadmill running, rotarod performance, grip strength, and gait analysis. Mice were then euthanized, and the left TA muscles were harvested for histological analysis.

### 4.3. Bioinformatics Analysis of scRNA-seq Data

ScRNA-seq datasets (GSE214892) were retrieved from the GEO database. The GSE214892 dataset was generated by pooling dissociated single cells from five mice per condition before scRNA-seq library construction. Therefore, each condition is represented by a single pooled sample. Individual cells were used as computational units for clustering and annotation but were not treated as independent biological replicates for statistical comparisons between conditions. Stringent quality control retained cells with 300–7000 detected genes, <20% mitochondrial content, and <3% erythrocyte transcripts. Data integration, normalization, and scaling were performed using the Seurat pipeline, with batch effects mitigated via Harmony. Cell clusters were identified through graph-based clustering and annotated using canonical markers. Intercellular communication was quantified using CellChat to infer ligand–receptor interaction strengths. Intracellularly, pathway activity scores and transcription factor (TF) regulon activities were deduced via PROGENy and the DoRothEA/VIPER framework, respectively. Gene Ontology enrichment analysis was performed using the enrichGO function in clusterProfiler (adjusted *p* < 0.05). Pseudotime trajectories were reconstructed using Monocle 2 with the DDRTree dimensionality-reduction algorithm, and genes showing dynamic expression patterns along the inferred pseudotime trajectory were identified.

### 4.4. Western Blotting

Cells were collected by a cell scraper. After centrifugation, the cell pellet was resuspended in RIPA buffer containing 1% PMSF and lysed on ice for 30 min. The lysate was then centrifuged at 12,000 rpm for 15 min at 4 °C, and the supernatant was collected. Protein concentration was determined using a BCA assay kit. Finally, loading buffer was added, and the samples were heated at 80 °C for 5 min. Proteins from each sample were separated by SDS-PAGE and transferred onto PVDF membranes (Merck Millipore, Darmstadt, Germany). The membranes were incubated with primary antibodies overnight at 4 °C. The following day, after another washing step, the membranes were incubated with HRP-conjugated secondary antibodies (Zenbio, China) for 1 h at room temperature. Finally, bands were visualized using an ECL substrate (Zenbio, China) and a Bio-Rad ChemiDoc imaging system. Data were analyzed using ImageJ software (version 1.54 P; NIH, Bethesda, MD, USA). For nuclear and cytoplasmic fractionation, proteins were extracted using a Nuclear and Cytoplasmic Protein Extraction Kit (Meilunbio, Dalian, China) according to the manufacturer’s instructions. Purity was confirmed by Western blotting using β-actin and Lamin A/C as cytoplasmic and nuclear markers, respectively.

### 4.5. Enzyme-Linked Immunosorbent Assay (ELISA)

The serially diluted standards and diluted test samples were added to the respective wells and incubated. After removal of unbound materials by washing, biotinylated detection antibody was added, followed by washing to remove unbound components, and then HRP-streptavidin (SABC) was added. After another wash, TMB substrate solution was added, and the reaction was terminated by adding stop solution. Absorbance was read at 450 nm using a Infinite 200 PRO microplate reader (Tecan, Männedorf, Switzerland).

### 4.6. Cell Viability Assay (CCK-8)

Cells were seeded into 96-well plates and cultured to the predetermined time points. They were then incubated with a mixture of Cell Counting Kit-8 (CCK-8) reagent (APExBIO, Houston, TX, USA) and DMEM medium (1:10 ratio) at 37 °C for 1 h. Subsequently, their viability was assessed by measuring the absorbance at 450 nm, and the results for the treatment groups were expressed as a percentage relative to the control group. Absorbance was read at 450 nm using a Infinite 200 PRO microplate reader (Tecan, Männedorf, Switzerland).

### 4.7. Immunofluorescence (IF) Staining

For cellular immunofluorescence, cells seeded on confocal dishes were fixed with 4% paraformaldehyde, permeabilized with 0.1% Triton X-100(Beyotime Biotechnology, Shanghai, China), and blocked with 5% serum. For tissue immunofluorescence, paraffin-embedded muscle sections were deparaffinized, rehydrated, and subjected to antigen retrieval before blocking. Both cells and tissue sections were then incubated with primary antibodies overnight at 4 °C, followed by fluorophore-conjugated secondary antibodies for 1 h at room temperature. Nuclei were counterstained with DAPI, and images were captured using a fluorescence microscope.

### 4.8. Wound Healing Assay

Cells were seeded into 6-well plates and cultured until they reached approximately 80–90% convergence. A sterile 200 μL pipette tip was then used to create a linear scratch vertically across each well. After scratching, the cells were gently washed with PBS to remove detached debris. Subsequently, the cells were cultured in serum-free medium for the indicated time periods. Finally, images were captured using a phase contrast microscope, and the migratory area were analyzed using ImageJ software (version 1.54 P; NIH, Bethesda, MD, USA).

### 4.9. Immunofluorescence Co-Localization

After treatment, cells were fixed with 4% paraformaldehyde for 15 min and washed with PBS. They were then permeabilized with Triton X-100 for 10 min, followed by PBS washes. To quench endogenous peroxidase activity, cells were incubated with 3% H_2_O_2_ solution for 15 min in the dark. After another PBS wash, cells were blocked with serum for 30 min at room temperature. Then the cells were incubated with primary antibodies overnight at 4 °C. Following three PBS washes, cells were incubated with HRP-conjugated secondary antibodies corresponding to the primary antibody host species for 50 min in the dark. Subsequently, cells were reacted with fluorescent dye for 10 min, washed with PBS, and then incubated with antibody stripping buffer for 10 min at 37 °C to remove primary-secondary antibody complex. For detection of second target, the entire staining procedure was repeated. Finally, nuclei were stained with DAPI, and after a final PBS wash, images were captured using a confocal microscope. Spatial overlap between MMP12 and SPP1 fluorescence signals was visualized using ImageJ (version 1.54 P; NIH, Bethesda, MD, USA). Because no replicate-level quantitative colocalization analysis was performed, the colocalization images were interpreted qualitatively and were not used for statistical inference.

### 4.10. Co-Immunoprecipitation (Co-IP)

Cells were harvested and lysed in RIPA buffer containing 1% PMSF for 1.5 h at 4 °C. After centrifugation at 14,000× *g* for 15 min, the supernatant was collected, with a portion reserved as the input control. To reduce non-specific binding, the lysate was pre-cleared with Protein A/G magnetic beads for 40 min. The pre-cleared supernatant was then partitioned and incubated overnight at 4 °C with either 2.5 µg of target-specific primary antibodies or control IgG. The following day, fresh Protein A/G beads were added to capture the immune complexes during a 4 h incubation at 4 °C. The beads were collected magnetically and washed sequentially with PBS-Tween 20, lysis buffer, and distilled water. Bound proteins were eluted by boiling the beads in 2 × SDS loading buffer at 100 °C for 10 min. The resulting eluates and input samples were subsequently analyzed via SDS-PAGE and Western blotting.

### 4.11. Flow Cytometry

Cells were collected, washed with PBS, and incubated with fluorophore-conjugated antibodies for 30 min at 4 °C in the dark. After washing to remove unbound antibodies, cells were resuspended in FACS buffer. Samples were acquired on a CytoFLEX flow cytometer (Beckman Coulter, Brea, CA, USA), and data were analyzed using FlowJo software (version 10.10, BD Biosciences, San Jose, CA, USA).

### 4.12. H&E Staining

Tissue sections were deparaffinized and rehydrated through graded ethanol. Sections were stained with hematoxylin for nuclear visualization, rinsed, and then counterstained with eosin for cytoplasmic and extracellular matrix components. After dehydration and clearing, slides were mounted with resinous medium and examined under a light microscope for morphological assessment.

### 4.13. Masson’s Trichrome Staining

Deparaffinized and rehydrated tissue sections were stained with Weigert’s iron hematoxylin for nuclei. Following washing, sections were treated with Biebrich scarlet-acid fuchsin solution for cytoplasmic and muscle fiber staining. After differentiation in phosphomolybdic–phosphotungstic acid solution, sections were incubated with aniline blue to stain collagen fibers. Slides were then dehydrated, cleared, and mounted for visualization of collagen deposition and muscle tissue architecture.

### 4.14. RNA Sequencing

Following digestion, washing, and centrifugation of L929 fibroblasts and C2C12 myoblasts that had received different treatments, TRIzol reagent was slowly added to the cells at a ratio of 5 × 10^6^ cells/mL. The mixture was then carefully transferred to RNase-free cryogenic tubes. Subsequently, the tubes were rapidly immersed in liquid nitrogen for 30 min to flash-freeze the samples. After freezing, the samples were removed from the liquid nitrogen, placed into self-sealing bags, and stored at −80 °C for long-term preservation. The samples were then sent to Majorbio Co. (Shanghai, China) for transcriptome sequencing. During the analysis, differentially expressed genes and enriched pathways were identified.

### 4.15. Treadmill Endurance Test

Each mouse was trained for 3 days before the formal experiment started. Then the mice were placed on a treadmill with an incline of 25°. The starting speed was set at 6 m per min. Every 2 min, the speed was gradually increased by 1 m per minute. The maximum speed was set as 22 m per minute. Mice were considered exhausted when they were shocked with electricity (0.8 mA) for five or more seconds, and the exercise endurance was evaluated by recording the time mice ran until exhausted.

### 4.16. Rotarod Test

Prior to the formal test, all animals underwent a period of acclimation. On the test day, each animal was placed on a swivel bar fatigue tester with a rotation speed at 4 rpm/min. The rotating speed was gradually increased by 8 rpm/min till the maximum speed reached 40 rpm/min. The time from the start of the rotation until the mouse fell from the rod onto the sensing platform below was recorded as the latency to fall. A cut-off time of 300 s was set to prevent injury from exhaustion.

### 4.17. Grip Strength Test

To assess hind limb grip strength, each mouse was gently held by the base of the tail, allowing its forepaws to rest on a platform. Then each mouse was lowered towards the bar until its hind paws gripped the bar securely. Once a firm grip was established, the tail of mouse was horizontally pulled away from the sensor in a smooth motion till the grip was released. The peak force at the moment of release was recorded in Newtons (N).

### 4.18. Gait Analysis

The Noldus CatWalk XT system was used for quantitative gait analysis to evaluate the locomotor function of mice. Before formal testing, mice were placed in the glass walkway to adapt to the environment. During the test, each mouse was placed at the aisle entrance and walked voluntarily along the glass aisle, with a high-speed Gigabit Ethernet camera capturing the locomotion data. The Walk Analysator software (version 10.6; Noldus Information Technology, Wageningen, The Netherlands) automatically screened and analyzed the footprints.

### 4.19. Statistical Analysis

Longitudinal functional data (gait, rotarod, treadmill, grip strength) were analyzed using a mixed-effects model in GraphPad Prism 8.0.2 (GraphPad Software, Inc., La Jolla, CA, USA). Treatment group, time, and group × time interaction were included as fixed effects, with each mouse treated as a random factor to account for repeated measurements within the same animal. Post hoc pairwise comparisons were performed within each time point (Day 3 and Day 14) using Tukey’s multiple comparisons test. For independent endpoint measurements, one-way ANOVA followed by Tukey’s post hoc test was used. All data are expressed as mean ± SEM. *n* refers to the number of independent animals per group. Significance levels are shown as * *p* < 0.05, ** *p* < 0.01, *** *p* < 0.001, **** *p* < 0.0001.

## 5. Conclusions

**Scheme 1:**
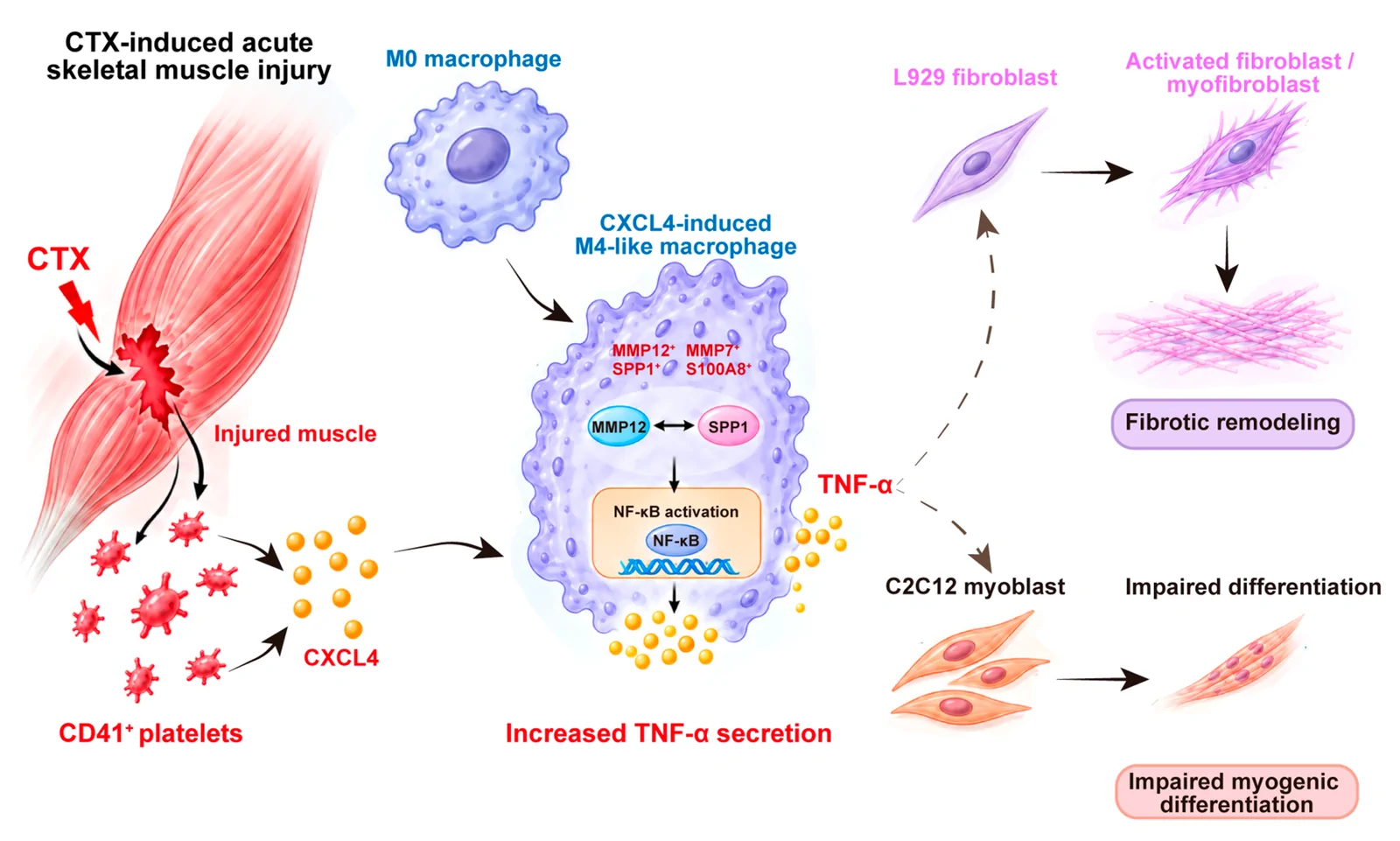
The proposed working model of the MMP12/SPP1/NF-κB signaling axis.

Using single-cell RNA sequencing, we observed an injury-induced *MMP12/SPP1*-positive macrophage subset with an M4-like transcriptional signature. Functionally, this subset is associated with fibrotic remodeling while impairing myoblast expansion and myogenic commitment. Mechanistically, M4-like macrophages acted through an MMP12-SPP1 signaling axis to activate NF-κB signaling and increase TNF-α secretion, which may contribute to divergent effects on fibroblasts and myoblasts. Collectively, our findings identify MMP12/SPP1/NF-κB signaling, with TNF-α as a potential downstream mediator, as a potential therapeutic target for limiting fibrosis and enhancing muscle regeneration after injury (Scheme 1).

## Data Availability

The data presented in this study are available on request from the corresponding author. The data are not publicly available due to ethical restrictions on animal research data.

## Supplementary Materials

The following supporting information can be downloaded at: https://www.mdpi.com/article/10.3390/ijms27209369/s1.

## Abbreviations

CTX	Cardiotoxin
ECM	Extracellular matrix
TNF-α	Tumor necrosis factor α
MHC	Myosin heavy chain
eMHC	embryonic Myosin heavy chain
MyoD	Myogenic differentiation
MyoG	Myogenin
