# Supplementary material for: M4-like Macrophages Drive Skeletal Muscle Fibrosis and Suppress Myogenesis via the MMP12/SPP1/NF-κB Axis
Source: Int J Mol Sci. 2026 Oct 21;27(20):9369. doi: 10.3390/ijms27209369 (PMC13704908; doi:10.3390/ijms27209369)
Supplement: Supplementary file 1 [file ijms-27-09369-s001.zip › ijms-4525879-supplementary.pdf]

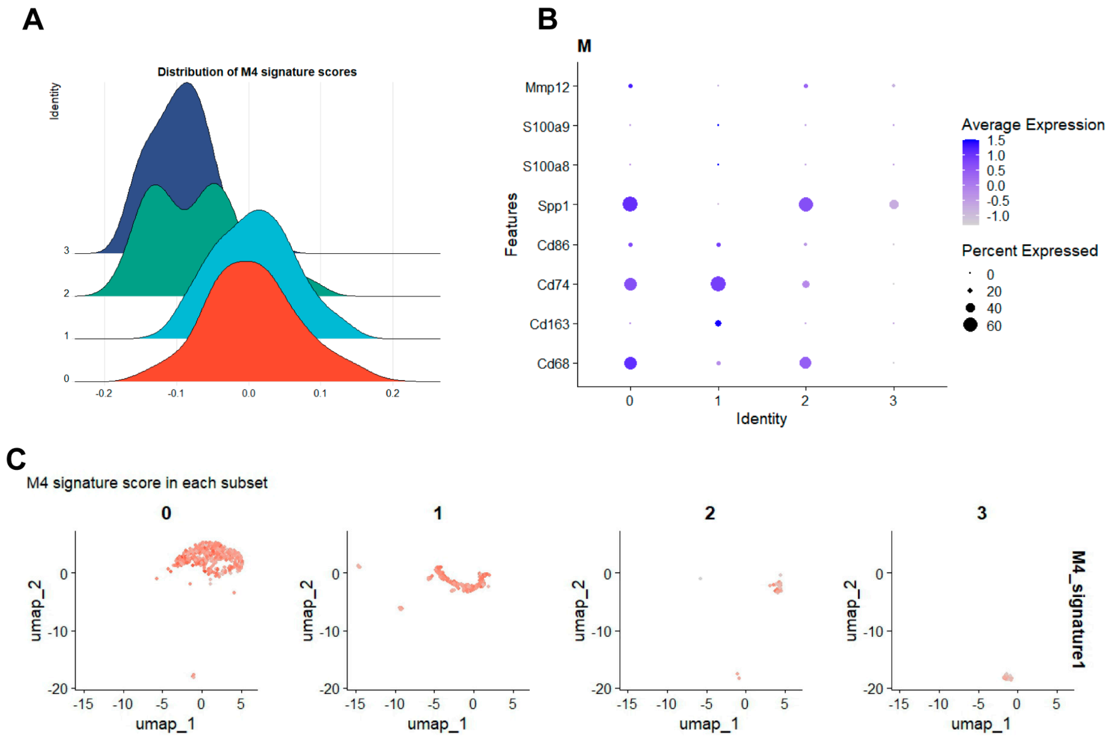

Figure S1:(A) Ridge plot showing the distribution of M4 macrophage signature scores across different cell cluster (cluster0, 1, 2, 3). (B) Dot plot showing the expression of selected M4 macrophage marker genes (MMP12, S100A9, SPP1, CD86, CD74, CD163, CD68) across cell cluster 0, 1, 2, and 3. (C) UMAP plots displaying the distribution of M4 macrophage signature scores within each cell subset (cluster 0, 1, 2, and 3).

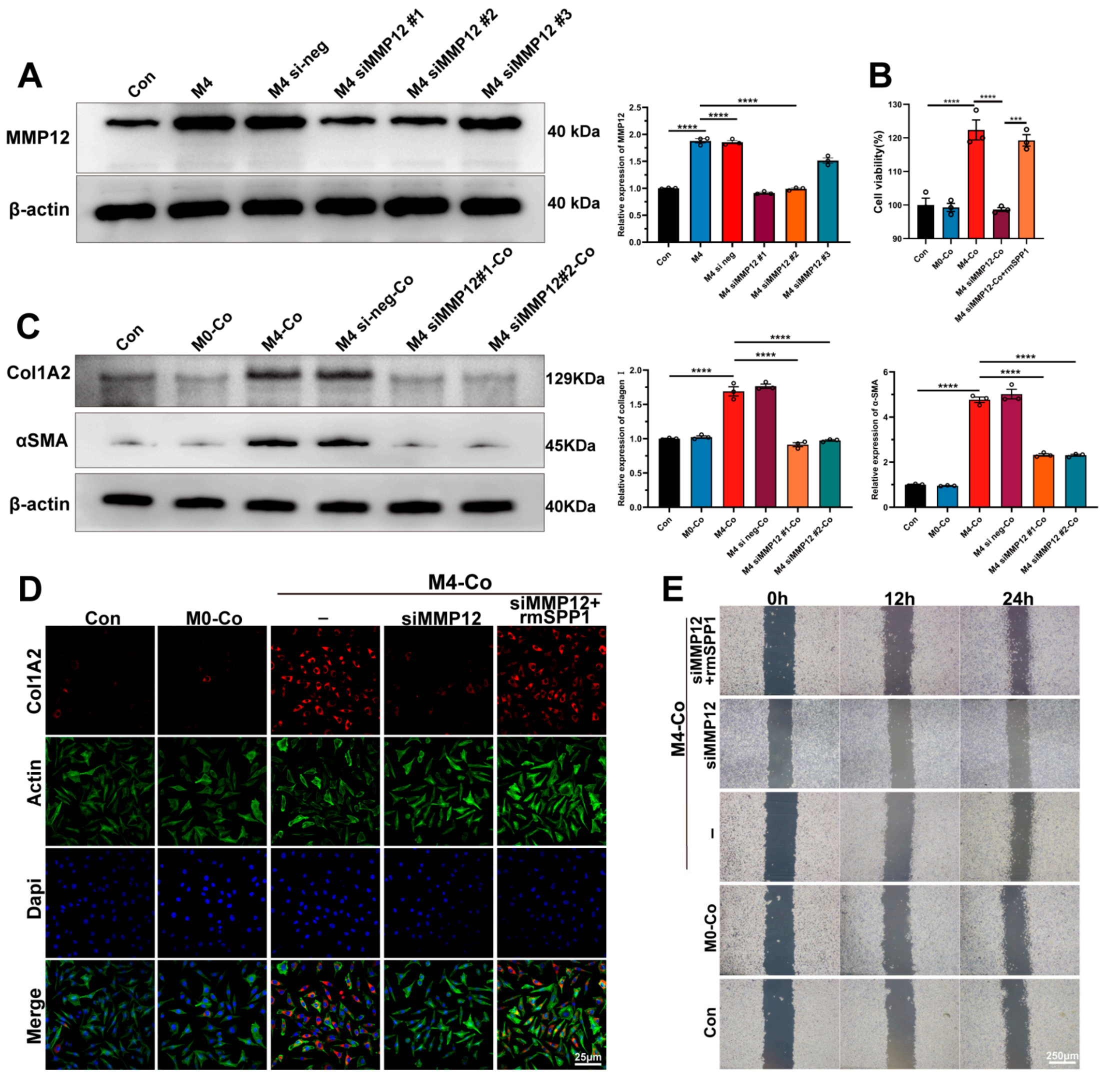

Figure S2: (A) Western blot validation of effects of siRNA sequences on MMP12 knock-down. SiMMP12#1 was chosen to minimize off-target effects in subsequent experiments. (B) Effect of M4-like macrophages transfected with siMMP12 and exogenous spp1 protein on L929 cell viability was detected using CCK-8 assay. (C) The expression of Col1A2 and αSMA of L929 cells following co-culture with M4-like macrophages or M4-like macrophages transfected with siMMP12 was detected by Western blot. (D) Representative fluorescence images of Col1A2 of L929 cells following co-culture with M4-like macrophages or M4-like macrophages transfected with siMMP12. Scale bar: 25 μm. (E) Effect of M4-like macrophages or M4-like macrophages transfected with siMMP12 on the migration of L929 cells. Representative images of wound healing assays were taken at 0, 12h and 24 h after scratching. Scale bar: 250 μm. *$P < 0.05$, **$P < 0.01$,***$P < 0.001$,****$P < 0.0001$.

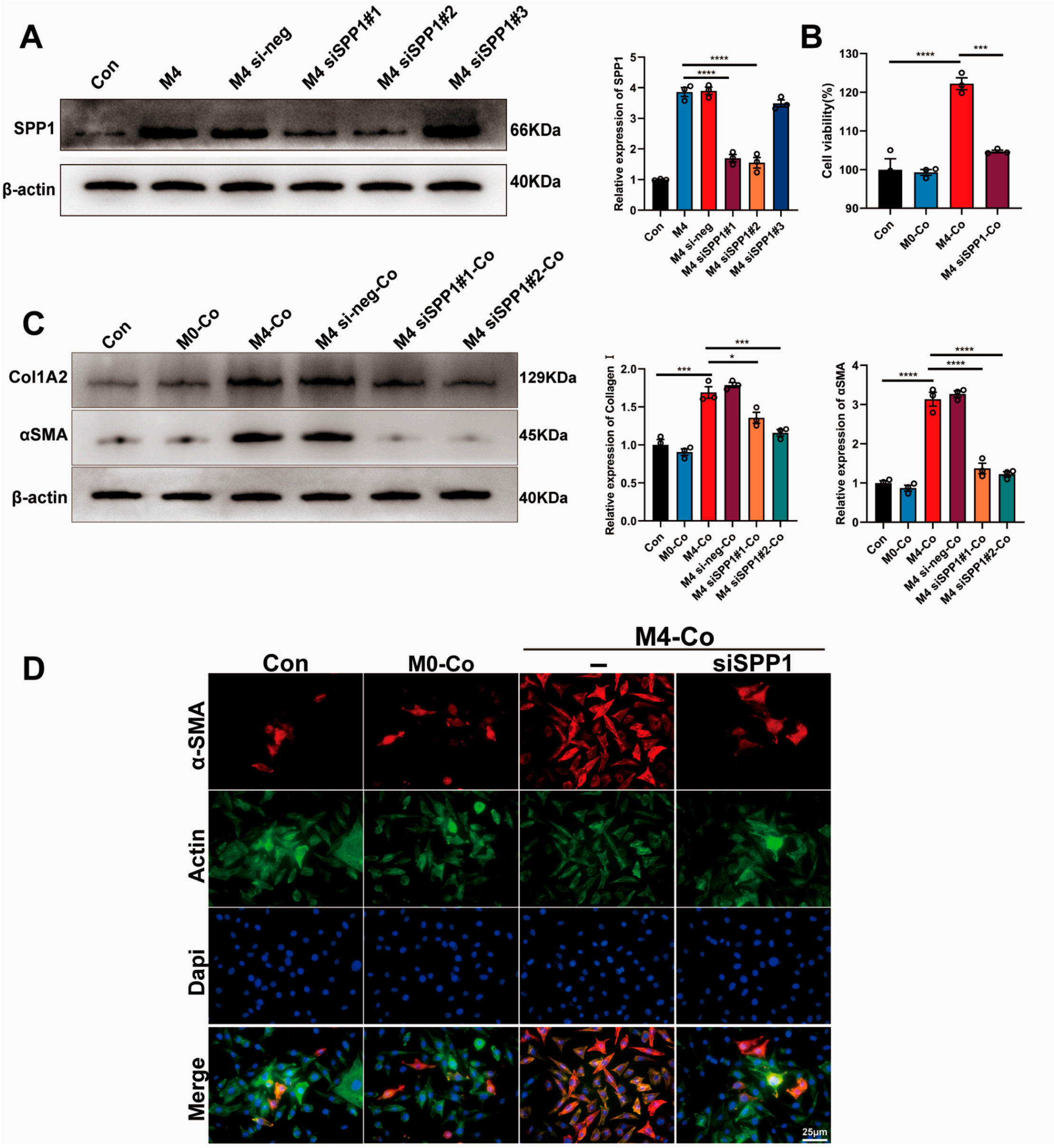

Figure S3: (A) Western blot validation of effects of siRNA sequences on SPP1 knock-down. SiSPP1#1 was chosen to minimize off-target effects in subsequent experiments. (B) Effect of M4-like macrophages and M4-like macrophages transfected with siSPP1 on L929 cell viability was detected using CCK-8 assay. (C) The expression of Col1A2 and αSMA of L929 cells following co-culture with M4-like macrophages or M4-like macrophages transfected with siSPP1 was detected by Western blot. (D) Representative fluorescence images of αSMA of L929 cells following co-culture with M4-like macrophages or M4-like macrophages transfected with siSPP1. Scale bar: 25 μm.

$^{*}P < 0.05$, $^{**}P < 0.01$,$^{***}P < 0.001$,$^{****}P < 0.0001$.

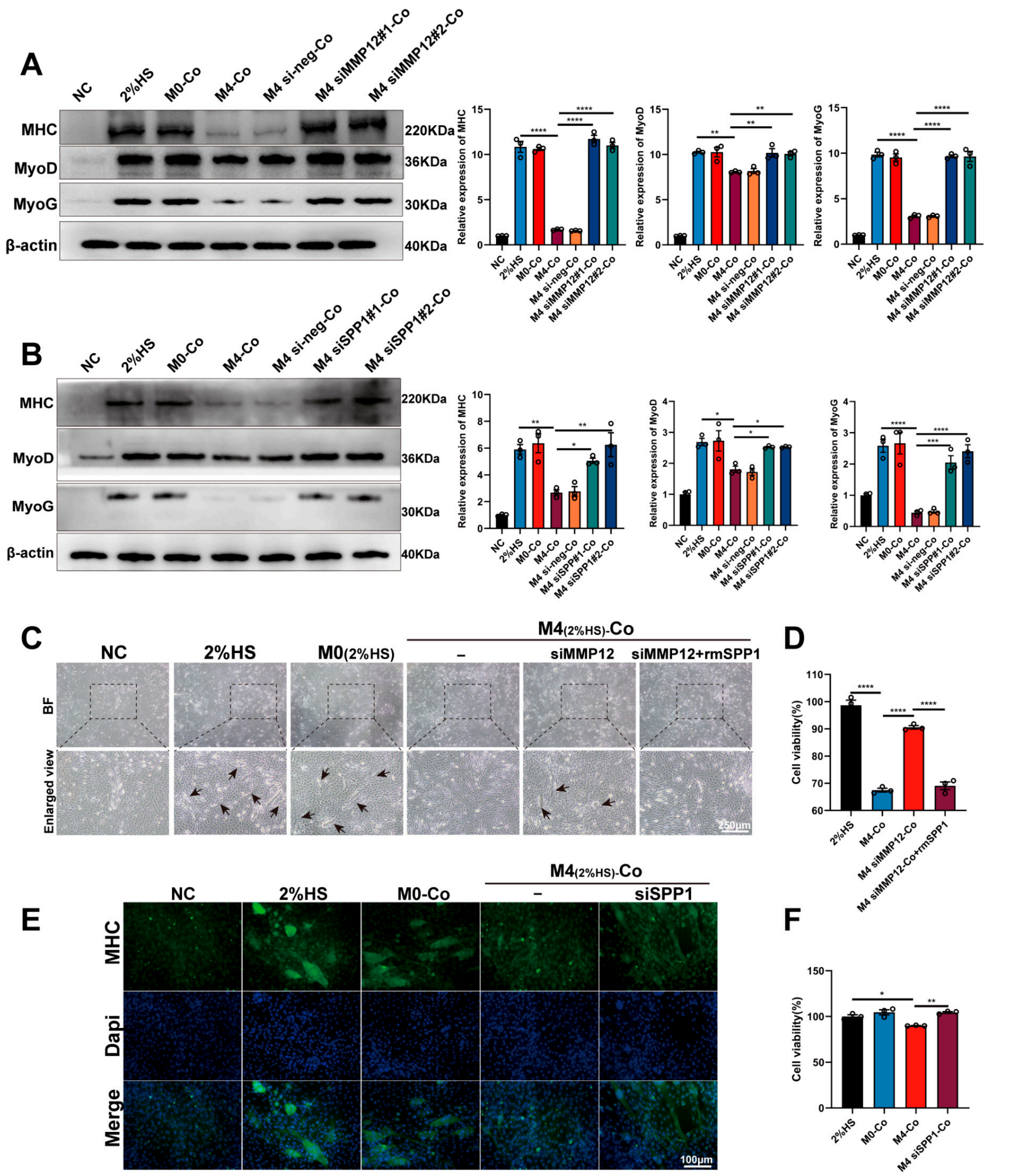

Figure S4: (A) Western blot validation of effect of MMP12-knockdown M4-like macrophages on C2C12 cells. SiMMP12#1 was used to minimize off-target effects in subsequent experiments. (B) Western blot validation of effect of SPP1-knockdown M4-like macrophages on C2C12 cells. SiSPP1#1 was used to minimize off-target effects in subsequent experiments. (C) Representative images of C2C12 cells differentiation after different co-culture. Black arrows indicate differentiated myotubes. Scale bar: 250μm. (D) Effect of M4-like macrophages and M4-like macrophages transfected with siMMP12 on C2C12 cell viability was tested using CCK-8 assay. (E) Representative

fluorescence images of MHC of C2C12 cells following co-culture with macrophages. Scale bar: 100μm. (F) Effect of M4-like macrophages and M4-like macrophages transfected with siSPP1 on C2C12 cell viability was tested using CCK-8 assay. *P < 0.05, **P < 0.01,***P < 0.001,****P < 0.0001.

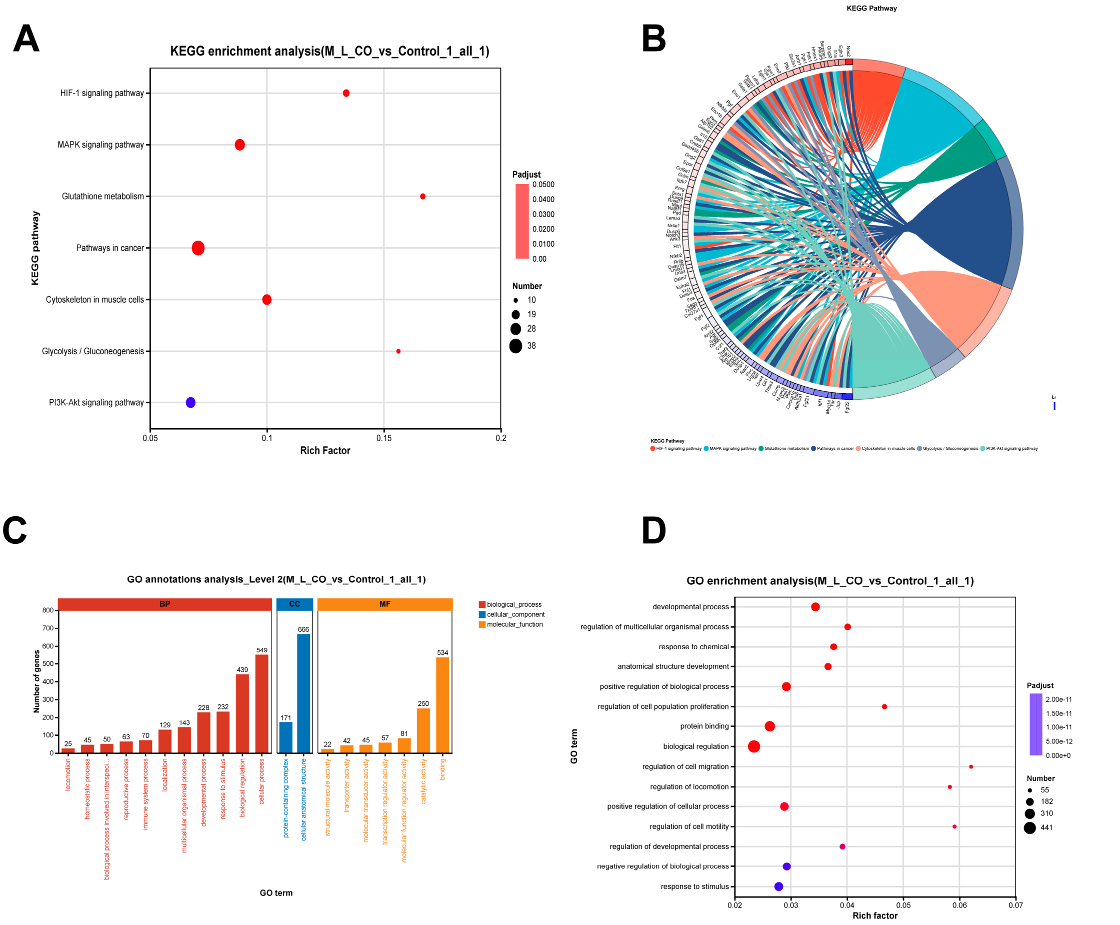

Figure S5: (A-B) KEGG enrichment analysis of L929 cells after co-culture with M4-like macrophages. (C-D) GO enrichment analysis of L929 cells after co-culture with M4-like macrophages.

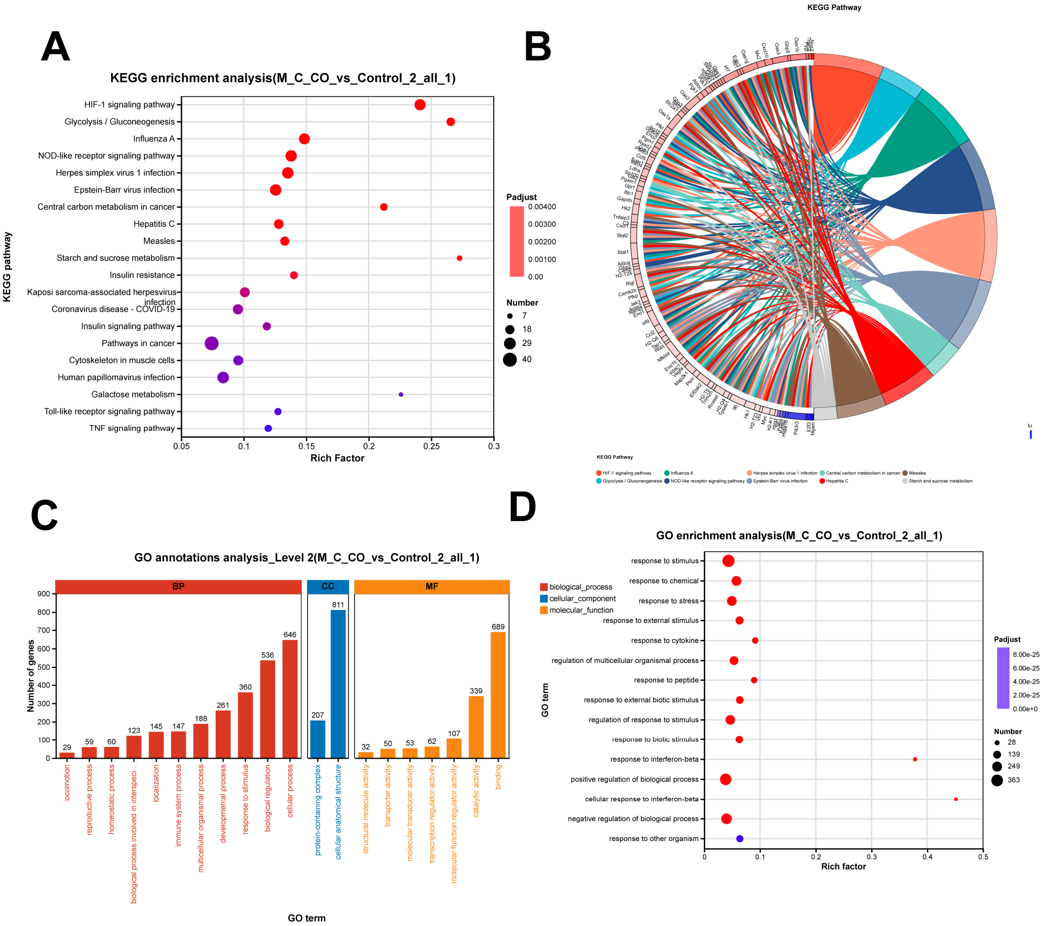

Figure S6:(A-B) KEGG enrichment analysis of C2C12 cells after co-culture with M4-like macrophages. (C-D) GO enrichment analysis of C2C12 cells after co-culture with M4-like macrophages.

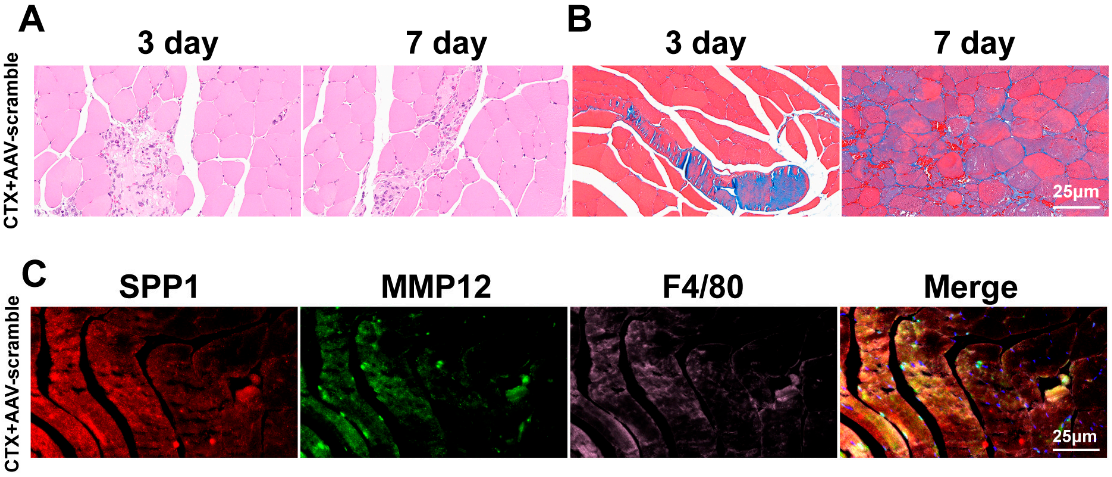

Figure S7:(A) H＆E stain of the tibialis anterior (TA) in the CTX+AAV-scramble group (n=3). Scale bar: 25μm. (B) The Masson stain of the tibialis anterior (TA) in the CTX+AAV-scramble group (n=3). Scale bar: 25μm. (C) The multiplex immunofluorescence staining of F4/80, SPP1, and MMP12 of the tibialis anterior (TA) in the CTX+AAV-scramble group. Scale bar: 25μm.
